# The influence of ferrocene anchoring method on the reactivity and stability of SBA-15-based catalysts in the degradation of ciprofloxacin *via* photo-Fenton process†

**DOI:** 10.1039/d3ra00188a

**Published:** 2023-03-14

**Authors:** Adrian Walkowiak, Lukasz Wolski, Maria Ziolek

**Affiliations:** a Adam Mickiewicz University, Poznań, Faculty of Chemistry Uniwersytetu Poznańskiego 8 61-614 Poznań Poland maria.ziolek@amu.edu.pl

## Abstract

The study is aimed at evaluation of the impact of ferrocene (Fc) anchoring method on the efficiency of its incorporation on the surface of mesoporous silica SBA-15, as well as the reactivity and stability of these hybrid organic–inorganic materials in degradation of ciprofloxacin (CIP) *via* photocatalytic, Fenton and photo-Fenton processes. For this purpose, Fc was anchored on SBA-15 supports *via* three different methods: (i) Schiff base formation, (ii) Friedel–Crafts alkylation, and (iii) click reaction (azide–alkyne cycloaddition). The as-prepared materials were characterized by powder X-ray diffraction, nitrogen physisorption, infrared spectroscopy and inductively coupled plasma optical emission spectrometry, as well as UV-visible and X-ray photoelectron spectroscopies. The highest efficiency of Fc anchoring was obtained when applying the Friedel–Crafts alkylation, while the least effective was the Schiff base formation. As concerns the catalysts activity, all materials exhibited negligible reactivity in the photocatalytic process, but were capable of degrading CIP in the presence of H_2_O_2_ (Fenton process). For all materials, the highest efficiency of CIP removal was observed for the photo-Fenton reaction. When expressed as the activity of a single Fc site, the most reactive were Fc species from the catalyst prepared by the click reaction. All materials, irrespectively of the ferrocene anchoring method, were deactivating over the reaction time because of Fc leaching. The highest stability in three subsequent reaction cycles was observed for the catalyst prepared by the azide–alkyne cycloaddition. Thus, the click reaction was found to be the best method for the preparation of Fc-containing catalysts for CIP degradation.

## Abstract 2

Click reaction is the most promising method for immobilization of ferrocene on SBA-15 for degradation of ciprofloxacin *via* photo-Fenton process.

## Graphical abstract

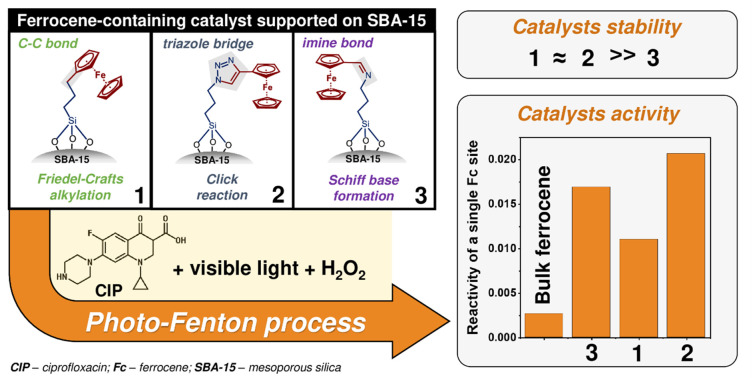

## 1. Introduction

In the face of the steadily growing population and increasing access to medical care, there is still a rising demand for antibiotics. Nowadays, these pharmaceuticals are used not only in human medicine but also as growth promoters, in agriculture, aquaculture, beekeeping, and livestock breeding.^1,2^ High global production and consumption of antibiotics entails severe environmental problems. According to very recent studies,^3^ significant amounts of various antibiotics have been detected not only in water treatment plants, but also in the natural water bodies, like rivers or lakes. This situation is alarming because the overconsumption of these pharmaceuticals and their increasing concentration in the environment, resulting from ineffective elimination of antibiotic pollutants from wastewater, can cause serious problems related to development of bacteria resistance to antibiotic treatment. In a long-term perspective, such a process may result not only in increasing morbidity, but also mortality.

One of the most commonly prescribed antibiotics are fluoroquinolones. From among this group of chemicals, ciprofloxacin (CIP) is the most common and represents 73% of their total consumption.^4^ It is used both in human and veterinary medicine to treat, *e.g.* skin and urinary tract infections.^4,5^ According to literature,^4,6^ CIP has been frequently detected in the natural water bodies and wastewater, and, for example, in hospital wastewater its concentration was found to reach up to 150 μg L^−1^. Therefore, development of effective methods for the removal of this antibiotic is an emerging environmental task which should be resolved, and which has been the focus of many researches and practitioners all over the world.

Advanced Oxidation Processes (AOPs) are a group of methods for wastewater treatment aiming at the formation of strongly oxidizing reactive oxygen species (ROS), for example, hydroxyl radicals (HO˙), superoxide radical anions (O_2_˙^−^), and hydroperoxyl radicals 
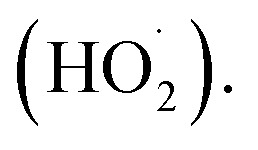
^2,7^ All the above-mentioned ROS exhibit high oxidizing potential and are capable of mineralization of selected hazardous organic contaminants, leading to the formation of non-hazardous inorganic molecules such as water, carbon dioxide, and inorganic salts.^2,7,8^ To date, many authors have reported that the efficient generation of highly active hydroxyl radicals can be reached in a facile Fenton process in which the reaction (1) between ferrous cations (Fe^2+^) and hydrogen peroxide leads to the formation of such radicals.^2,9^ Many authors have also established that the efficiency of Fenton reaction in generating hydroxyl radicals can be significantly accelerated after the exposure of the reaction media to an external light source.^9,10^ Such a process is called a photo-Fenton reaction.^9^ The positive impact of light on the efficiency of hydroxyl radicals formation *via* Fenton process is usually considered in terms of more efficient reduction of Fe^3+^ ions to Fe^2+^*via* reaction (2) which is known as the key limiting step of the overall Fenton process.^9^

1 Fe^2+^ + H_2_O_2_ → Fe^3+^ + HO˙ + OH^−^

2 Fe^3+^ + H_2_O_2_ + *hν* → Fe^2+^ + HOO˙ + H^+^

**Fig. 1:**
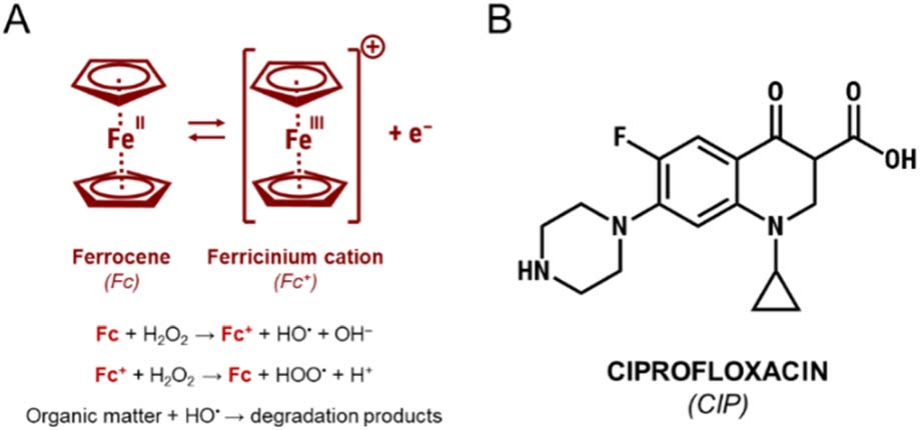
(A) Ferrocene-catalyzed activation of hydrogen peroxide in the Fenton process; (B) structural formula of ciprofloxacin, a model antibiotic pollutant.

One of the sources of ferrous cations used in Fenton process is ferrocene (Fc) (Fig. 1). It is an organometallic compound that consists of two cyclopentadienyl rings and one Fe^2+^ cation that form a sandwich-like π-bonding complex with a [Fe(η^5^-C_5_H_5_)_2_] formula.^11^ Ferrocene is stable in aqueous solutions and almost insoluble in water.^12^ Importantly, it is characterized by a very strong light absorption in the UV-vis range and a good redox reversibility due to the presence of Fe^II^/Fe^III^ conjugated redox pair (see Fig. 1).^11,12^

Recently, much effort has been undertaken to anchor Fc on the surface of various solid supports like graphitic carbon nitride,^13,14^ conjugated porous polymers,^15,16^ metal–organic frameworks,^17,18^ clays,^19^ and silica gels.^20^ According to these previous reports,^19,21^ Fc loaded on various supports is more active than bulk ferrocene, and is a promising candidate for designing of novel heterogeneous visible-light-driven photo-Fenton systems addressed to degradation of organic pollutants in wastewater. However, in the majority of the above-mentioned studies, Fc was anchored to the surface of solids *via* Schiff base formation between ferrocenecarboxyaldehyde and a support containing amine groups.^14,16,21,22^ To the best of our knowledge, fundamental studies concerning the impact of the ferrocene anchoring method on the activity and stability of such hybrid organic–inorganic materials in photo-Fenton processes are sparse, especially in terms of degradation of antibiotics. Therefore, this study focuses on filling this gap. In our study Fc is supported on ordered mesoporous silica SBA-15 by the three procedures, namely: (i) Schiff base formation, (ii) Friedel–Crafts alkylation, and (iii) click reaction (azide–alkyne cycloaddition), and the reactivity and stability of the as-prepared hybrid materials in CIP degradation *via* photocatalytic, Fenton and photo-assisted Fenton processes is assessed. SBA-15 was selected as a support for ferrocene because of its high stability under acidic conditions in which Fenton process is known to be the most efficient.^9^ Moreover, SBA-15 is characterized by large surface area, high concentration of surface hydroxyl groups as well as well-defined porosity that may enable obtainment of a high dispersion of Fc on the surface of this support.^23^

**Fig. 2:**
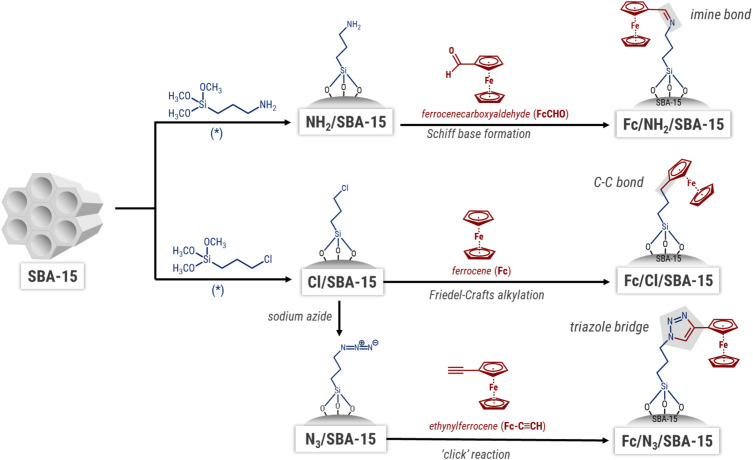
The procedures of ferrocene anchoring used in this work.

The base of the work hypothesis was that depending on the nature of silica functionalization agent used for Fc anchoring, this active component may show different stabilities depending on various strength of the bonds between Fc and a given functionalizing agent. Moreover, it was expected that various electron densities of the active component, induced by electronic interaction with the functionalizing agent, may have significant influence on the redox properties of iron species in the anchored ferrocene, and thus, on their catalytic activity. Thus, three different preparation procedures for preparation of hybrid materials containing Fc, illustrated in Fig. 2 and above-mentioned, were applied in this study. In the first method, amino-organosilane was used for the functionalization of SBA-15, and Schiff base formation was expected as a result of the reaction between the surface amine group of the functionalizing agent and ferrocenecarboxyaldehyde (FcCHO). The second procedure assumed functionalization of the SBA-15 support with chloro-organosilane followed by coupling with ferrocene *via* the Friedel–Crafts alkylation. In the third procedure, SBA-15 was firstly functionalized with chloro-organosilane. Then, Cl species from the chloro-organosilane were substituted by azide *via* reaction with sodium azide. Finally, ferrocene was anchored on the surface of the as-obtained material *via* triazole ring formed as a result of the click reaction between ethynylferrocene and surface azide species.

## 2. Experimental

### 2.1 Chemicals and reagents

The chemicals used in this work are the following: Pluronic P123 (Sigma Aldrich), tetraethyl orthosilicate (TEOS) (Sigma Aldrich, 99%), (3-aminopropyl)trimethoxysilane (APTMS) (Sigma Aldrich, ≥97%), (3-chloropropyl)trimethoxysilane (CPTMS) (Sigma Aldrich, ≥97%), sodium azide (Fisher Chemical, ≥99%), ferrocene (Sigma Aldrich, 98%), ferrocenecarboxyaldehyde (Sigma Aldrich, 98%), ethynylferrocene (Angene, 98%), toluene (Stanlab, pure p.a.), absolute ethanol (CHEMPUR, 99.8%), dimethylformamide (DMF) (Fisher Chemical, ≥99%), *tert*-butyl alcohol (^*t*^BuOH) (Alfa Aesar, 99%), 2-propanol (iPrOH) (Stanlab, pure p.a.), copper(ii) sulfate pentahydrate (Sigma Aldrich, ≥99%), sodium ascorbate (Sigma Aldrich, ≥98%), zinc chloride (Alfa Aesar, ≥98%, anhydrous), glacial acetic acid (CHEMPUR, 99.9%), hydrogen peroxide (Sigma Aldrich, 25–35% w/w in H_2_O, for ultratrace analysis). Deionized water was used throughout the experiments.

### 2.2 Synthesis of catalysts

#### 2.2.1 Preparation of mesoporous silica (SBA-15)

In a typical SBA-15 synthesis, Pluronic P123 (16 g) was dissolved in a 0.7 M aqueous solution of hydrochloric acid (700 g). After the surfactant dissolution, the temperature was kept at 35–40 °C, and TEOS (34.1 g) was added dropwise upon continuous stirring. Following 20 h of agitation at the same temperature, the mixture was transferred to an oven and heated at 100 °C for 24 h. The solid product formed was then separated by filtration, washed with 1200 mL of deionized water and dried overnight at room temperature. Finally, the residual soft template (Pluronic P123) was removed by calcination at 500 °C for 8 h (heating rate: 1 °C min^−1^). Before any further modification of SBA-15, the siliceous support was dried at 100 °C for 18 h in order to remove water residue.

#### 2.2.2 Modification of SBA-15 with organosilanes

SBA-15 was functionalized by the grafting procedure described elsewhere.^24^ In a typical post-synthesis modification, dry SBA-15 support was stirred in a round-bottom flask containing toluene (20 mL of toluene per 1 g of SBA-15 support). Then, (3-aminopropyl)trimethoxysilane was added (2.5 mL of APTMS per 1 g of SBA-15) and the resulting mixture was refluxed for 18 h at approximately 100 °C. The solid sample was then separated by filtration, and washed with toluene (150 mL), deionized water (100 mL) and acetonitrile (30 mL). Finally, the solid product was dried overnight at 80 °C. The as-obtained material was labelled as NH_2_/SBA-15.

A similar procedure was used to functionalize SBA-15 support with (3-chloropropyl)trimethoxysilane (CPTMS). In this case, 2.5 mL of the chlorosilane were used per 1 g of silica support. The obtained material was labelled as Cl/SBA-15.

In order to introduce azide moieties onto the surface of SBA-15, the previously synthesized Cl/SBA-15 sample was subjected to the nucleophilic substitution of Cl atoms with azide groups. For this purpose, 2.5 g of the obtained Cl/SBA-15 material were stirred in 100 mL of dimethylformamide. Next, sodium azide (0.244 g) was added and the resulting mixture was heated for 18 h at 80 °C upon continuous stirring (400 rpm). The obtained product was separated by filtration and washed with water (500 mL). Finally, the solid was dried overnight at 80 °C. The resulting material was labelled as N_3_/SBA-15.

### 2.3 Anchoring of ferrocene species on the functionalized SBA-15 supports

In this study, ferrocene was anchored to SBA-15 supports *via* three different ways: (i) Schiff base formation, (ii) Friedel–Crafts alkylation, and (iii) azide–alkyne cycloaddition (click reaction). All experimental details are given below.

#### 2.3.1 Schiff base formation

In a typical procedure, 3 g of amine-modified SBA-15 (*i.e.* NH_2_/SBA-15 sample) were dispersed in 75 mL of absolute ethanol in a round bottom flask. Then, 2.25 mmol of ferrocenecarboxyaldehyde and a few droplets of glacial acetic acid were added upon continuous stirring. Following 20 h of heating under reflux (∼80 °C), the brownish solid formed was collected by centrifugation, and washed several times with small portions of ethanol and water until the supernatant was completely colorless. The as-obtained material was then dried overnight at 60 °C. The resulting catalyst of a pale brown color was labelled as Fc/NH_2_/SBA-15.

#### 2.3.2 Friedel–Crafts alkylation

In a typical synthesis route, 3 g of SBA-15 modified with chlorosilane (*i.e.* Cl/SBA-15 sample) were dispersed in 200 mL of dimethylformamide (DMF) in a round bottom flask. Then 2.25 mmol of ferrocene and anhydrous zinc chloride (4 molar equivalents in relation to ferrocene) were introduced to the suspension upon continuous stirring. The resulting mixture was then agitated (400 rpm) under reflux (*ca.* 150–160 °C) for 20 h. Upon reaching the boiling point of DMF, the reaction mixture turned black. The dark brown solid formed in the reaction was collected by filtration, washed with ethanol (0.5 L) and water (1.5 L). The as-obtained sample was dried overnight at 60 °C. The resulting catalyst was labelled as Fc/Cl/SBA-15.

#### 2.3.3 Azide–alkyne cycloaddition (click reaction)

In a typical procedure, azide-modified sample (N_3_/SBA-15; 2 g) was stirred in a mixture consisting of 150 mL solvent (water : *tert*-butyl alcohol, 1 : 1 volume ratio) and 1.5 mmol ethynylferrocene, resulting in formation of a yellow suspension. Then, aqueous solution of copper(ii) sulfate (source of copper for the click reaction, 0.1 equivalent) and sodium ascorbate (reducing agent used for *in situ* generation of Cu^+^ ions by reduction of Cu^2+^ species) were added to the above suspension upon continuous stirring. After this step, the mixture turned orange. The resulting suspension was agitated intensively (1000 rpm) at room temperature for 20 h. The solid formed was then separated by filtration, and washed with water (500 mL) and dried overnight at 60 °C. The resulting catalyst was labelled as Fc/N_3_/SBA-15.

### 2.4 Characterization of materials

The iron, zinc and copper ion contents in the catalysts were determined using the inductively coupled plasma optical emission spectrometry (ICP-OES) method (ICP-OES SPECTRO BLUE TI). The digestion of the samples was performed using a Microwave Digestion System (Multiwave-PRO, Anton Paar, Austria).

The XRD patterns were recorded on a D8 Advance diffractometer (Bruker) using Cu Kα radiation (*λ* = 0.154 nm), with a step size of 0.05° in the 2*θ* range of 6–60°.

N_2_ adsorption–desorption isotherms were obtained at −196 °C using an ASAP 2020 Physisorption Analyzer (Micromeritics, USA). Before measurements, the samples were degassed at 120 °C for 10 h. The specific surface area of the materials obtained was calculated by the Brunauer–Emmett–Teller (BET) method, and the average pore size was estimated based on the Density Functional Theory (DFT).

The morphology of the synthesized catalysts was investigated using a field-emission scanning electron microscope (FE-SEM) Quanta 250 FEG, FEI operating at the accelerating voltage of 10 kV. Energy dispersive X-ray analysis (EDX) and EDX mapping were performed using the EDS analyzer and beam accelerating voltage of 30 kV. All measurements were conducted on carbon adhesive conductive tape, without metallization.

The concentration of functionalization agents anchored to the surface of all SBA-15 supports was estimated on the basis of elemental analysis using a FLASH 2000 elemental analyzer (Thermo Scientific).

The FTIR spectra of the samples were acquired in the range of 4000 to 400 cm^−1^ (resolution 4 cm^−1^, number of scans = 64) using a Vertex 70 spectrometer (Bruker, Germany). For FTIR measurements with KBr, the samples were dispersed in KBr pellet (2 mg of the sample and 200 mg of KBr).

Diffuse reflectance UV-vis spectra (DR UV-vis) were recorded on a Varian Cary 300 Scan spectrophotometer equipped with a diffuse reflectance accessory. The spectra were recorded at room temperature from 200 to 800 nm using Spectralon as the reference. Only in the case of bulk ferrocene, the powdered sample was diluted with mesoporous silica (SBA-15; Fc : SBA-15 weight ratio = 1 : 10) prior to DR UV-vis measurements. Dilution of bulk ferrocene was necessary due to its very strong light absorption both in UV and visible region.

X-ray photoelectron spectroscopy (XPS) was performed using an ultra-high vacuum photoelectron spectrometer based on a Phoibos 150 NAP analyzer (Specs, Germany). The analysis chamber was operated under vacuum with a pressure close to 5 × 10^−9^ mbar and the sample was irradiated with a monochromatic Al Kα (1486.6 eV) radiation. Any charging that might occur during the measurements (due to incomplete neutralization of ejected surface electrons) was accounted for by rigidly shifting the entire spectrum by a distance needed to set the binding energy of the C 1s assigned to adventitious carbon to the assumed value of 284.8 eV.

### 2.5 Catalytic tests

Catalytic activity of the materials obtained was tested in degradation of ciprofloxacin as a model antibiotic *via* photocatalytic, Fenton and photo-Fenton processes. All the reactions were carried out in a glass reactor (EasyMax 102 Advanced Thermostat System, Mettler-Toledo). In a typical reaction, 30 mg of the catalyst were dispersed in 80 mL of CIP aqueous solution (15 mg L^−1^; pH ∼ 3.0 adjusted with sulfuric acid). In the case of Fenton process, the reaction was performed in the dark and was initiated by addition of 50 μL of H_2_O_2_ (30%). In the photocatalytic process, no hydrogen peroxide was used and the reaction mixture was irradiated from the top with 200 W Hg–Xe lamp (Hamamatsu LC8 Spot Light, model: L9566-06A) equipped with a UV filter (transmissive light above 400 nm only) and UV-light guide (model: A10014-50-0110). For the photo-Fenton process, the reaction was performed both in the presence of H_2_O_2_ and upon its exposure to visible light. In all reactions, the antibiotic degradation was monitored by means of UV-vis spectroscopy (Varian, Cary 300 UV-vis spectrophotometer). For this purpose, after the proper time, 4 mL of the reaction mixture were collected, the catalyst was separated from the solution by filtration through a 0.2 μm Millipore filter, and the absorption spectrum of the filtrate was recorded using an UV-vis spectrophotometer in the range from 190 to 800 nm. The efficiency of CIP removal was calculated using the following equation: CIP removal [%] = ((*C*_0_ − *C*_*t*_)/*C*_0_) × 100%, where *C*_0_ is the initial concentration of CIP and *C*_*t*_ is the concentration of CIP after a given time (*t*) of the reaction. The role of hydroxyl radicals in photo-Fenton process was investigated by addition of an appropriate scavenger (*i.e.* 2-propanol; concentration of the scavenger in reaction mixture: 10 mmol L^−1^). For the reuse tests, the catalyst was separated from the reaction mixture by filtration, washed with water and ethanol, and dried overnight at 60 °C. Then, the dry catalyst was used in a subsequent reaction cycle without any additional treatment.

## 3. Results and discussion

### 3.1 Characteristic of the materials

The mesoporous silica, hexagonally ordered SBA-15, was used as a support for ferrocene. Since Fc cannot be directly attached to silica surface, three different functionalization methods were applied in order to introduce proper functional groups on silica surface prior to Fc loading: (i) grafting with (3-aminopropyl)trimethoxysilane (support labelled as NH_2_/SBA-15), (ii) grafting with (3-chloropropyl)trimethoxysilane (support labelled as Cl/SBA-15), and (iii) grafting with (3-chloropropyl)trimethoxysilane followed by nucleophilic substitution with sodium azide (support labelled as N_3_/SBA-15). All ferrocene-containing materials used in this study are schematically shown in Fig. 2.

Table 1 presents the amount of ferrocene (expressed as the wt% of iron and the efficiency of Fc loading) introduced to the previously functionalized materials. The highest efficiency of Fc anchoring was observed for the catalyst prepared *via* the second procedure (Friedel–Crafts alkylation), whereas the lowest – for the sample functionalized by (3-aminopropyl)trimethoxysilane in which the Fc was anchored by the Schiff base formation, *i.e.* the method most frequently used for Fc attachment to the solid surfaces.^14,16,21,22^ One can expect that the efficiency of Fc immobilization was related to the amount of functionalizing agent introduced to SBA-15 and/or the yield of the reaction between the functionalizing agent and the ferrocene source. As implied by Table 1 data, the concentration of functionalizing agent has no obvious influence on the efficiency of ferrocene anchoring. For instance, SBA-15 functionalized with (3-aminopropyl)trimethoxysilane contained the greatest amount of the functionalizing agent but this material exhibited the lowest efficiency of Fc loading. Thus, it was concluded that the main process limiting the efficiency of ferrocene immobilization on SBA-15 was the yield of the reaction between the ferrocene source and the functionalizing agent.

**Table 1:** Content of elements in the catalysts

Sample	Fe^a^ (wt%)	Zn^a^ (wt%)	Cu^a^ (wt%)	Efficiency of Fc introduction (%)	N^b^ (wt%)	N-containing functional groups (mmol g^−1^)	N-containing functional groups to ferrocene molar ratio
Fc/NH_2_/SBA-15	0.46	<0.01	<0.01	11.4	1.68	1.20	14.6
Fc/Cl/SBA-15	1.58	0.03	<0.01	39.3	—	—	—
Fc/N_3_/SBA-15	0.78	<0.01	0.17	19.4	1.78	0.42	3.0

a Established using ICP-OES.

b Established using elemental analysis.

It is important to point out that all catalysts contained small amounts of zinc and copper species which originated from various metal salts used during anchoring of Fc on SBA-15 supports (Table 1). As concerns Zn, its amount in all materials was so small that it can be omitted in the further consideration of the catalysts activity in CIP removal. The same is true for the Cu content in Fc/Cl/SBA-15 and Fc/NH_2_/SBA-15 materials. In the catalyst containing the greatest amount of copper species (Fc/N_3_/SBA-15, Table 1), a possible contribution of Cu^2+^ ions to degradation of CIP will be discussed in depth in further part of the article. However, there is no doubt that iron species are predominant over Cu species in this material. Taking into account molecular weights of both metals and their wt% in Fc/N_3_/SBA-15 sample (Table 1), it can be estimated that this material contains approximately 5.2 times more moles of iron species than copper ones (*ca.* 14% of copper moles in the total moles of Fe + Cu).

**Fig. 3:**
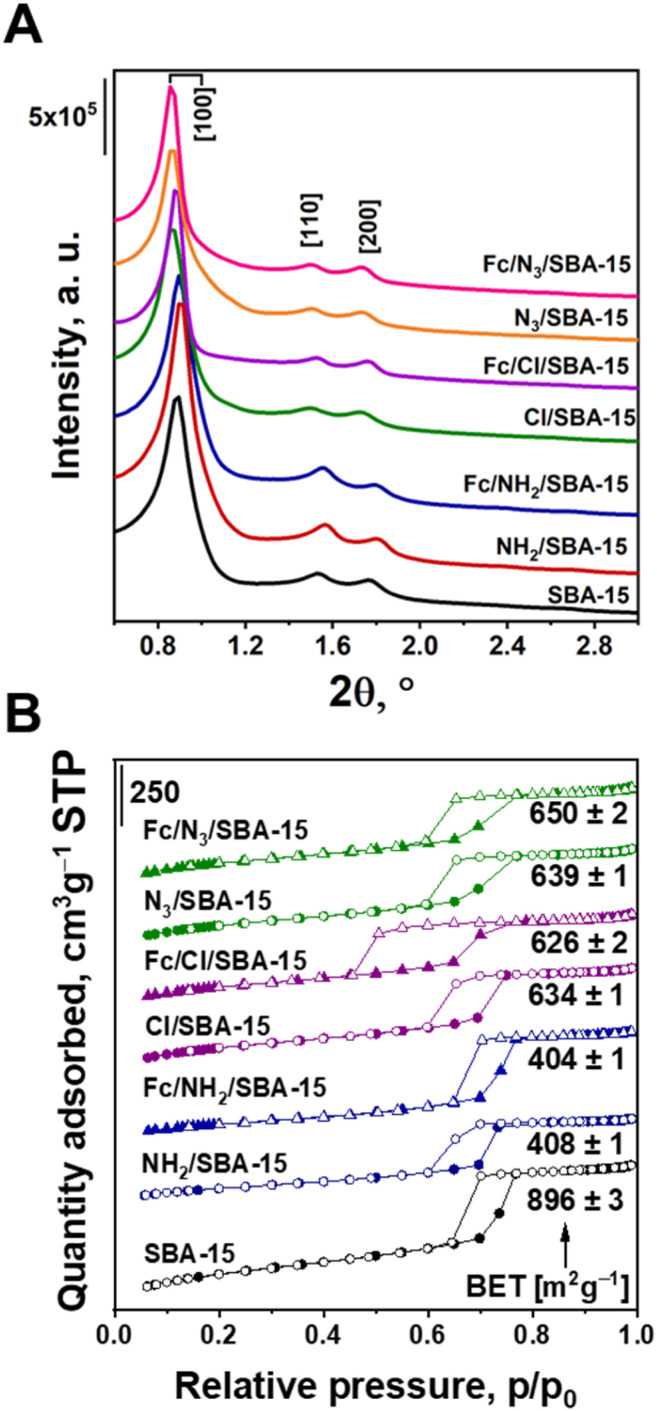
(A) Small-angle range of X-ray diffractograms of the prepared materials; (B) low-temperature N_2_ adsorption–desorption isotherms and BET surface areas of the examined samples.

As concerns the structure of mesopores silica, the multistep modification of SBA-15 applied in this work could lead to the disordering or destruction of this siliceous material. However, XRD patterns shown in Fig. 3A clearly indicate that hexagonal ordering of silica was preserved in all synthesized catalysts, as evidenced by the presence of three reflections at *ca.* 0.9, 1.6 and 1.8°, corresponding to the Miller indices [100], [110] and [200], respectively.^25,26^ The most intensive [100] peak is typical of hexagonally ordered mesopores in SBA-15 structure, while the less intensive two other reflections indicate that these pores are well-ordered in a long range. Slight shifts in the position of [100] peak are caused by partial blockade of mesopores by the organosilane modifiers, and a decrease in the average pore diameter.

This feature is well illustrated by the nitrogen physisorption isotherms (Fig. 3B) and pore size distribution (Fig. S1†). All the isotherms are of type IV according to IUPAC classification,^27^ and are typical of SBA-15 materials. Thus, nitrogen physisorption data further confirm that the structure of pristine SBA-15 was preserved after anchoring of organosilanes and ferrocene. The BET surface areas and shape of hysteresis loops were, however, influenced by the method of Fc anchoring. From among all functionalized supports, the most pronounced decrease in BET surface area was observed for NH_2_/SBA-15 (Fig. 3B). As implied by Table 1 data, this material contained three times greater amount of the amino-organosilane than azide moieties content in N_3_/SBA-15. Thus, one can clearly conclude that the most pronounced decrease in BET surface area of this material originated from the highest efficiency of APTMS anchoring and was associated with blockage of pores by the anchored organosilane. Similar phenomenon was observed in numerous previous studies.^28^ As concerns the Fe-containing materials, the highest efficiency of Fc immobilization (deduced from the amount of iron in the samples) was observed in Fc/Cl/SBA-15 (Table 1). This sample discloses the most pronounced hysteresis loop, characteristic of nitrogen desorption from ink-bottle-shaped pores.^27^ Thus, Fc attaching to the functionalized SBA-15 also caused partial blockage of the mesopores, and as a consequence, the pore size distribution exhibits two systems of mesopores (Fig. S1†). This phenomenon is strictly dependent on the amount of Fc introduced, and is less pronounced for the samples with lower Fc loading.

**Fig. 4:**
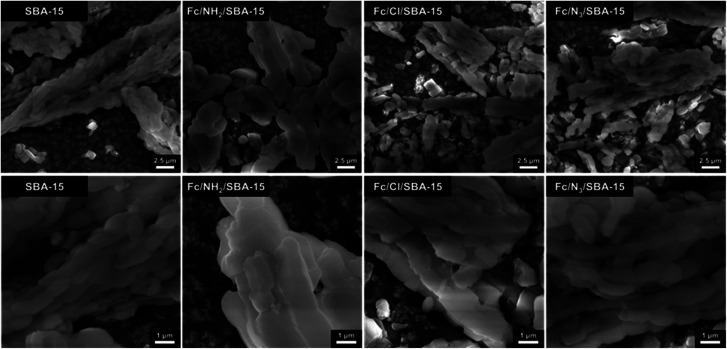
SEM images of pristine SBA-15 and Fc-containing catalysts shown at different magnification.

Morphology of the materials was investigated by means of scanning electron microscopy (SEM). Fc/N_3_/SBA-15 and Fc/Cl/SBA-15 catalysts exhibited similar morphology to the pristine SBA-15 but some clearly noticeable changes in the size and shape of the particles of siliceous support were observed for Fc/NH_2_/SBA-15 (Fig. 4), for which the highest efficiency of organosilane anchoring was noticed (Table 1). In the case of this material, aggregates of SBA-15 particles were much shorter and thicker (Fig. 4). This indicates that the most pronounced decrease in BET surface area observed for this catalyst resulted not only from partial blockage of pores by anchored organosilane but also from changes in morphology of the siliceous support after functionalization with APTMS. Indeed, as shown in Fig. S2,† morphology of SBA-15 has been already changed after functionalization with APTMS, and further anchoring of Fc did not have any significant impact on morphology of the functionalized support (compare Fig. 4 and S2†). Thus, SEM data are in a good agreement with those obtained from XRD and nitrogen physisorption. Dispersion of Fc species on the surface of SBA-15 was determined by SEM-EDS. As revealed by elemental mapping shown in Fig. S3–S6,† iron species were homogenously dispersed on the surface of all Fc-containing catalysts. In view of this observation, one can state that ferrocene in all catalysts existed in the form of finely dispersed Fc species anchored on SBA-15 support, rather than large aggregates of bulk Fc.

**Fig. 5:**
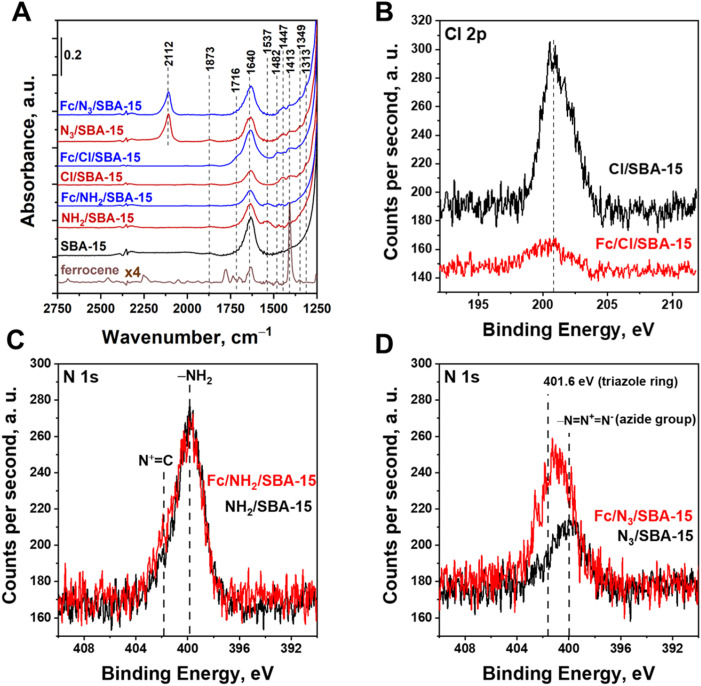
(A) FTIR spectra of the prepared materials; (B) XPS spectra of Cl/SBA-15 and Fc/Cl/SBA-15 in the Cl 2p range; (C) XPS spectra of NH_2_/SBA-15 and Fc/NH_2_/SBA-15 in the N 1s range; (D) XPS spectra of N_3_/SBA-15 and Fc/N_3_/SBA-15 in the N 1s range.

Successful loading of the modifiers on SBA-15 was also confirmed by the FTIR and XPS (Fig. 5). As concerns the IR data, the most representative is the region 2250–1300 cm^−1^ in which only one IR band typical of O–H vibrations in the adsorbed water molecules is observed for SBA-15 support.^25,29^ According to literature,^30–32^ this band is overlapped by the band originating from N–H deformation vibrations in amine groups. Thus, the presence of amine groups could not be clearly concluded based only on IR data. Nevertheless, it was confirmed by XPS analysis. As shown in Fig. 5C, in the XPS spectra recorded for NH_2_/SBA-15 one can observe a well pronounced N 1s peak at approximately 399.8 eV which is characteristic of nitrogen in amine groups.^33^ Deposition of ferrocene on NH_2_/SBA-15 support did not result in any clearly noticeable changes in FTIR spectra (Fig. 5A). The lack of such changes in the IR spectra resulted more likely from a very low concentration of ferrocene species when compared to the overall amount of amine groups (see Table 1). However, some changes in the properties of nitrogen species after anchoring of Fc were observed by XPS (Fig. 5C). According to literature,^34^ formation of a Schiff base could be followed by the appearance of an additional N 1s peak typical of imine species, which is shifted by approximately 0.6 eV relative to the amine peak. Since only a very small amount of all amine species were consumed during the Fc anchoring, and the peak characteristic of imine is overlapped with that characteristic of the pristine amine groups, thus, the spectral component confirming the formation of a Schiff base cannot be easily resolved by analyzing this region. However, the formation of N=C bonds in Fc/NH_2_/SBA-15 sample was indirectly confirmed by the appearance of another spectral component at approximately of 401.6 eV that is assigned to X-ray-oxidized nitrogen in N^+^=C species^34^ (see Fig. 5C). In the IR spectrum of Cl/SBA-15, there are two IR bands at approximately 1447 and 1413 cm^−1^ that are related to the presence of (3-chloropropyl)trimethoxysilane modifier anchored on SBA-15 support^35^ (Fig. 5A). The presence of this chloro-organosilane in Cl/SBA-15 sample was also confirmed by the XPS data (see Fig. 5B). Interestingly, after Fc anchoring to the chlorine-functionalized support, the intensity of the XPS peak characteristic of chlorine was significantly reduced (Fig. 5B), which was associated with the appearance of a new IR band at 1482 cm^−1^ assigned to ferrocene (Fig. 5A). At the same time, a significant increase in the intensity of another IR band typical of Fc, located at approximately 1640 cm^−1^, was also observed. Thus, combined results of XPS and IR studies clearly indicated that the elimination of HCl from chloro-organosilane occurs in parallel with the Friedel–Crafts alkylation leading to anchoring of Fc *via* C–C bond. The IR spectrum of N_3_/SBA-15 presents a well-visible band at ∼2112 cm^−1^ which is characteristic of –N=N^+^=N^−^ vibration in azide species.^25,32^ The presence of azide species in N_3_/SBA-15 sample was also confirmed by XPS. As shown in Fig. 5D, in the XPS spectra recorded for the N 1s region there is a broad peak at approximately 400.0 eV which is assigned to N_3_ species.^36^ As implied by FTIR data, anchoring of ferrocene to N_3_/SBA-15 support resulted in a slight decrease in intensity of the IR band typical of azide species (Fig. 5A), indicating the consumption of azide species during the anchoring of Fc, which was associated with well-pronounced changes in the N 1s region of the XPS data. As shown in Fig. 5D, after the ferrocene anchoring a new peak at approximately 401.6 eV appeared, indicating successful formation of triazole ring.^37^

**Fig. 6:**
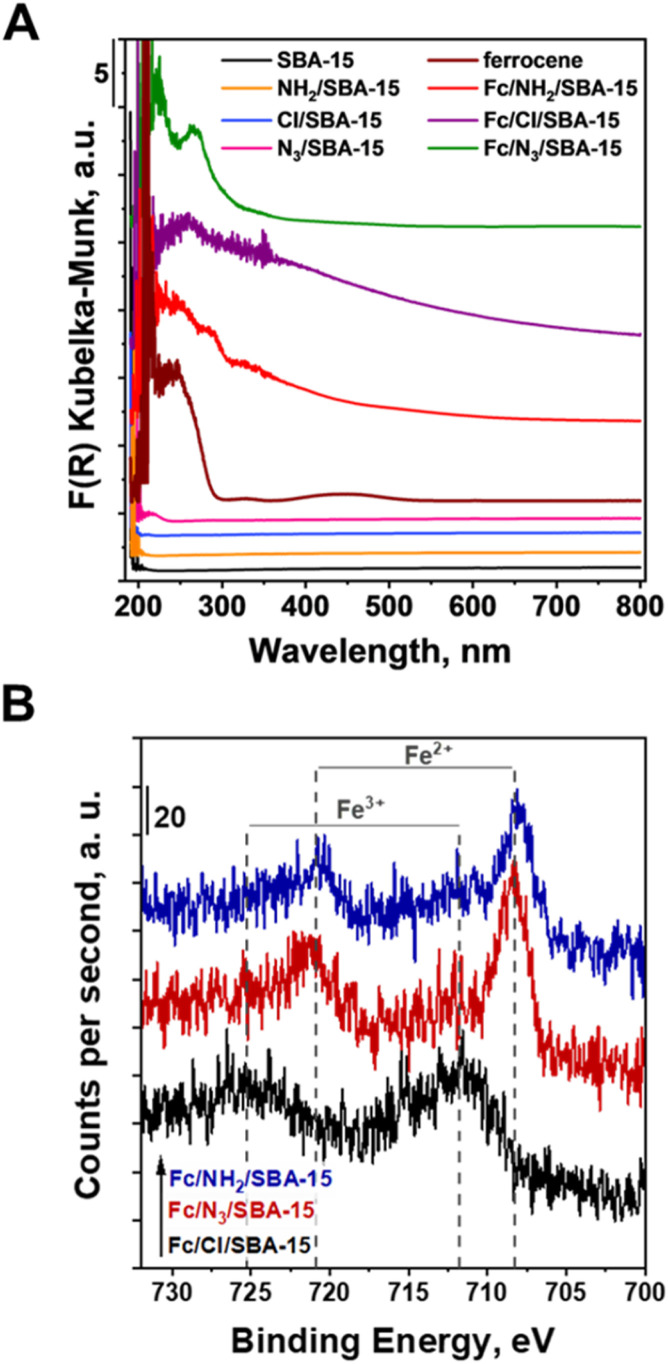
(A) Diffuse reflectance UV-vis spectra of the examined materials and ferrocene; (B) XPS spectra of the Fc-containing materials in the Fe 2p range.

Efficient loading of Fc on the surface of SBA-15 supports was also confirmed by DR UV-vis spectroscopy. As shown in Fig. 6A, SBA-15 support does not absorb visible light. In contrast to the parent silica support, all ferrocene-containing materials exhibited efficient absorption of light both in ultraviolet (UV) and visible (vis) region. Similar absorption bands were also observed for bulk ferrocene. Thus, DR UV-vis studies further confirmed that ferrocene was successfully anchored to the surface of all materials prepared in this study. Interestingly, all ferrocene-containing catalysts supported on SBA-15 had slightly different optical properties, especially in the UV region. For instance, an absorption band at approximately 275 nm was observed only in the spectrum of Fc/N_3_/SBA-15. According to literature, this absorption band is attributed to formation of triazole species.^38^ Thus, anchoring of Fc *via* formation of triazole ring, concluded form XPS data, was additionally confirmed by the UV-vis studies. Moreover, in the DR UV-vis spectra recorded for Fc/NH_2_/SBA-15, one can observe additional absorption bands at approximately 250 and 300 nm which are assigned to the p–p* and n–p* transitions of the –C=N group in Schiff base modified imine.^34,39^ Thus, DR UV-vis studies further confirmed that Fc was anchored in different ways to the surface of all SBA-15-based catalysts.

Differences in the optical properties of ferrocene species anchored in different ways to SBA-15 supports were further confirmed by XPS. As shown in Fig. 6B, in the region Fe 2p in the XPS spectra of Fc/NH_2_-SBA-15 and Fc/N_3_-SBA-15 one can find two Fe 2p peaks at the binding energies of approximately 708.3 and 720.9 eV that are assigned to Fe^2+^ ions in ferrocene (Fe 2p_3/2_ and Fe 2p_1/2_ spin orbitals, respectively).^40^ Interestingly, in the spectrum of Fc/N_3_/SBA-15, these peaks were shifted toward higher binding energy values, indicating lower electron density in the neighborhood of iron species in Fc/N_3_/SBA-15, when compared to that in the sample prepared by the Schiff base formation. It further confirms different ways of Fc anchoring to the surface of these two materials. Totally different electronic properties of iron species were observed for Fc/Cl/SBA-15 (Fig. 6). Instead of the two above-mentioned peaks typical of Fe^2+^ species in ferrocene, one can distinguish only two well-resolved extra peaks at binding energies of approximately 711.8 and 725.2 eV, respectively, assigned to Fe^3+^ species in ferrocene.^41^ Thus, XPS and UV-vis data clearly indicated that Fc deposition method had a significant impact both on the optical and electronic properties of the ferrocene species anchored to SBA-15 supports, and further confirmed that the ferrocene species were anchored in different ways on the surface of all materials used in this study.

### 3.2 Catalytic activity and stability of materials in CIP degradation

#### 3.2.1 Reactivity of Fc-containing materials

**Fig. 7:**
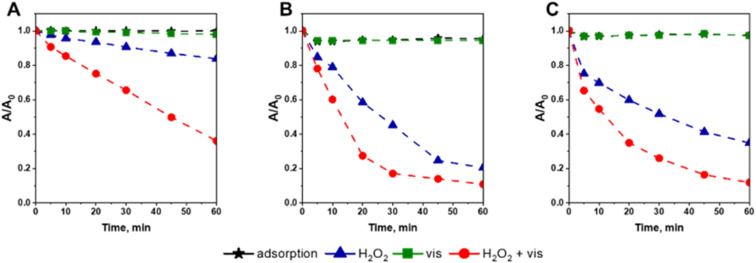
Efficiency of CIP degradation *via* photocatalytic, Fenton, and photo-Fenton processes in the presence of (A) Fc/NH_2_/SBA-15, (B) Fc/Cl/SBA-15, and (C) Fc/N_3_/SBA-15. Reaction conditions: 30 mg of the catalyst, 80 mL of CIP solution (15 mg L^−1^, pH = 3), 50 μL of H_2_O_2_ (30%), stirring rate: 600 rpm.

Fig. 7 shows the results of CIP degradation *via* photocatalytic, Fenton, and photo-Fenton process in the presence of Fc-containing catalysts prepared in this study. According to the results, all catalysts, irrespectively of Fc deposition method, exhibited negligible activity in CIP degradation under visible light irradiation. It clearly showed that Fc-containing catalysts do not exhibit any noticeable photocatalytic activity in CIP degradation. However, all materials were capable of degrading the antibiotic in the presence of hydrogen peroxide. As shown in Fig. 7, their reactivity in Fenton process was found to be strongly correlated to the efficiency of ferrocene anchoring. The higher the Fc content in the SBA-15-based materials, the higher the catalysts activity in CIP degradation. Thus, the most active was the material prepared by the Friedel–Crafts reaction (Fc/Cl/SBA-15), while the lowest activity was observed for the material in which Fc was anchored through the Schiff base formation (Fc/NH_2_/SBA-15). In the presence of all catalysts, the antibiotic degradation proceeded according to the pseudo-first order kinetics (see Fig. S7†). The estimated rate constants (*k*) were of 0.0029, 0.0261 and 0.0150 min^−1^ for Fc/NH_2_/SBA-15, Fc/Cl/SBA-15, Fc/N_3_/SBA-15, respectively (Fig. S7†). In agreement with previous reports in this field,^14,16,19^ the efficiency of Fenton process was greatly improved when the reaction mixture was additionally exposed to visible light (Fig. 7). Similarly to the Fenton process, the highest efficiency in antibiotic removal *via* photo-Fenton reaction was observed for the catalyst with the highest ferrocene loading (Fig. 7). It is important to emphasize that all reference samples, including pristine SBA-15 support and SBA-15 grafted with different functionalizing agents, exhibited negligible activity in CIP removal (Fig. S8†). It shows that SBA-15 support and modifiers themselves did not exhibit any activity in H_2_O_2_ activation. Slight decrease in CIP concentration, observed in the presence of SBA-15, resulted mainly from the antibiotic adsorption on the siliceous support, rather than its degradation (Fig. S8†). This hypothesis was confirmed by the fast decrease in the antibiotic concentration at the beginning of the process and negligible changes in CIP concentration after longer reaction times. As far as the reactivity of the samples containing Fc in photo-assisted Fenton process is concerned, it is important to underline that after the exposure of the reaction mixture to visible light the reaction rate constants (*k*) increased from 0.0029, 0.0261, and 0.0150 min^−1^ to 0.0130, 0.0629 and 0.0377 min^−1^ for Fc/NH_2_/SBA-15, Fc/Cl/SBA-15 and Fc/N_3_/SBA-15, respectively (Fig. S9†). In view of these observations, one can clearly conclude that the exposure of reaction media to visible light may enhance catalytic activity of all Fc-containing materials in the Fenton process but has no influence on the reaction mechanism. In other words, the degradation reaction may be accelerated by external light source, but cannot be directed into different pathways. It is in agreement with low photocatalytic activity of Fc-containing nanomaterials, and indicates that the enhanced reactivity in the photo-Fenton reaction resulted most probably from more efficient light-induced reduction of ferricinium cations (Fc^+^) to ferrocene (Fc) that facilitated more efficient production of hydroxyl radicals *via* the Fenton process (Fc + H_2_O_2_ → Fc^+^ + HO˙ + OH^−^; Fig. 1A), rather than degradation of CIP *via* additional ROS formed in a conventional photocatalytic process.^12,19^ Transformation of ferricinium cations (Fc^+^) to ferrocene (Fc) is known as the rate-determining step in activating of H_2_O_2_ over Fc-containing catalysts, and reduction of Fc^+^ into Fc may be accelerated upon exposure to external light source.^12,16,19,20^ Thus, our hypothesis about more efficient regeneration of Fc species *via* reaction Fc^+^ + H_2_O_2_ → Fc + HOO˙ + H^+^ (Fig. 1A) in the presence of visible light is highly probable. Similar mechanism of H_2_O_2_ activation in photo-Fenton process *via* electron transfer from Fc sites to H_2_O_2_ was proposed in several previous studies (*e.g.* ref. 19).

**Fig. 8:**
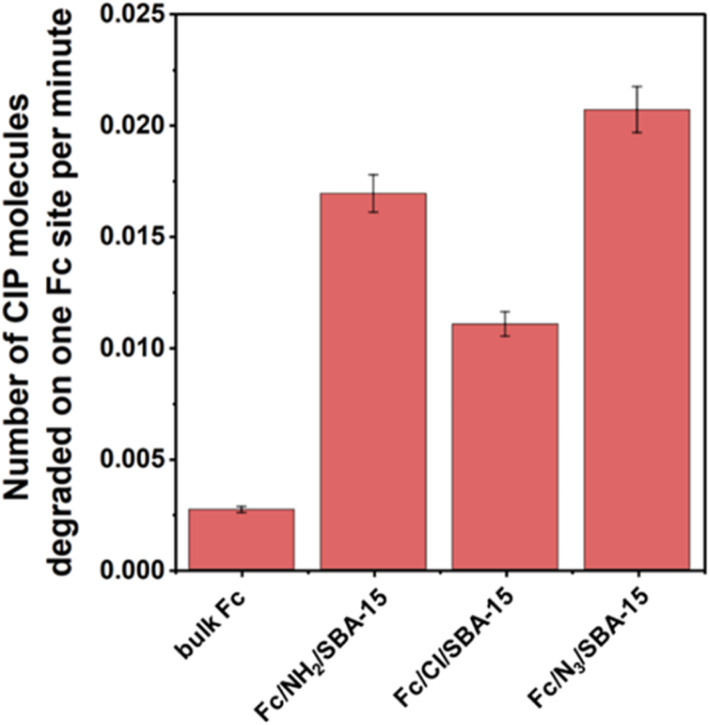
Normalized efficiency of CIP degradation in the presence of Fc-containing catalysts after 30 min of the photo-Fenton process expressed as number of CIP molecules degraded by ROS formed on a single Fc site per time unit. Reaction conditions: 30 mg of the catalyst, 80 mL of CIP solution (15 mg L^−1^, pH = 3), 50 μL of H_2_O_2_ (30%), irradiation with visible light (*λ* > 400 nm), stirring rate: 600 rpm. In the case of bulk ferrocene, mass of the catalyst used was reduced to 5 mg.

Direct comparison of catalysts reactivities in the photo-Fenton process, is however, slightly problematic because of different Fc loadings (Table 1). For comparison purposes, the reactivities of all materials were normalized and expressed as moles of CIP molecules degraded by ROS formed on a single Fc site per time unit (Fig. 8). Contribution of copper species to the degradation of CIP in photo-Fenton process was totally neglected in these calculations, since these metal ions were leached from the catalysts during the reaction (Table S1†), and Cu^2+^ ions themselves were found to be inactive in the photo-Fenton process (see Fig. S10†). As shown in Fig. 8, ferrocene species from all catalysts prepared in this work significantly outperformed bulk ferrocene. From among all materials, the highest reactivity of a single Fc site was observed for the sample prepared by the click reaction (Fc/N_3_/SBA-15). The reactivity of Fc in the samples prepared by the Schiff base formation was only slightly lower than that established for the material in which ferrocene was immobilized by the formation of triazole ring (Fig. 8). The least efficient CIP removal was observed for a single Fc site in the catalyst prepared by Friedel–Crafts alkylation. As concerns the lowest activity of ferrocene in Fc/Cl/SBA-15, many previous reports indicated that chloride ions may react with hydroxyl radicals leading to formation of Cl˙^−^ which are less reactive and exhibit lower oxidation potential. Since Fc/Cl/SBA-15 catalyst contains chloride ions, one can expect that the lowest reactivity of chlorine-containing sample resulted mainly from the scavenging effect of chloride ions.^42^ The above-described differences in the reactivity of Fc species in various samples may also result, to some extent, from the contribution of electronic effects. Only in the case of Fc/N_3_/SBA-15, ferrocene was anchored by formation of triazole ring that has aromatic nature and may influence the electron density in the neighborhood of iron ions, and thus, the redox reactivity of iron species by electron withdrawing effect. Indeed, as shown in Fig. 6B, the binding energy of Fe 2p peaks in Fc/N_3_/SBA-15 was shifted toward higher energy values than that observed for Fc/NH_2_/SBA-15 sample, indicating the presence of strong electronic interaction between ferrocene and triazole ring that resulted in a decrease in electron density in the neighborhood of iron species.

**Fig. 9:**
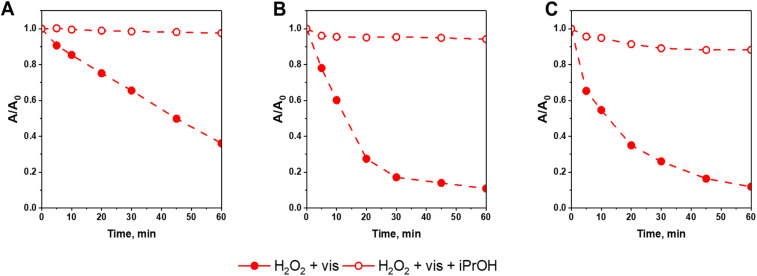
Influence of 2-propanol (iPrOH, 10 mmol L^−1^) on the kinetics of CIP degradation *via* photo-Fenton process in the presence of (A) Fc/NH_2_/SBA-15, (B) Fc/Cl/SBA-15, and (C) Fc/N_3_/SBA-15. Reaction conditions: 30 mg of the catalyst, 80 mL of CIP solution (15 mg L^−1^, pH = 3), 50 μL of H_2_O_2_ (30%), irradiation with visible light (*λ* > 400 nm), stirring rate: 600 rpm.

Results obtained in this work clearly show that the positive impact of light on the efficiency of a Fenton-like process may be considered mainly in terms of enhancing the rate of ferricinium cations (Fc^+^) reduction to ferrocene (Fc) which are the key species responsible for activating H_2_O_2_ to hydroxyl radicals. In order to further confirm this conclusion and shed more light on the mechanism of the photo-Fenton process, additional catalytic tests in the presence of 2-propanol as hydroxyl radical scavenger were performed. As shown in Fig. 9, the reactivity of all catalysts in CIP degradation was almost totally quenched in the presence of 2-propanol, confirming that hydroxyl radicals formed *via* the Fenton process were the key oxidizing agents responsible for efficient degradation of the antibiotic. It is also important to underline that the efficiency of quenching effect was the same for all materials prepared in this study. It further confirmed that the Fc deposition method on SBA-15 support has no influence on the mechanism of H_2_O_2_ activation both in the absence and presence of external light source.

#### 3.2.2 Stability of Fc-containing materials

**Fig. 10:**
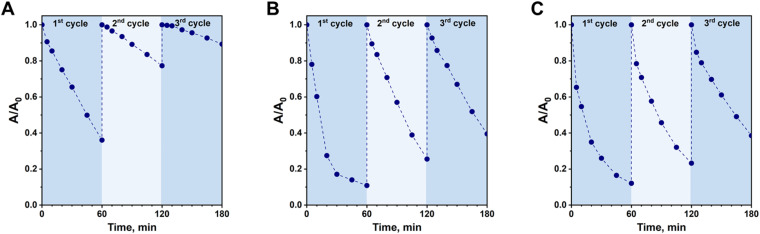
Efficiency of CIP degradation during three subsequent reaction cycles in the presence of (A) Fc/NH_2_/SBA-15, (B) Fc/Cl/SBA-15, and (C) Fc/N_3_/SBA-15. Reaction conditions: 30 mg of the catalyst, 80 mL of CIP solution (15 mg L^−1^, pH = 3), 50 μL of H_2_O_2_ (30%), irradiation with visible light (*λ* > 400 nm), stirring rate: 600 rpm.

Stability of heterogeneous catalysts is one of the key factors limiting their potential application in wastewater remediation. In this work, the stability of Fc-containing catalysts prepared by various methods was tested in three subsequent reaction cycles. As shown in Fig. 10, the lowest stability in the photo-Fenton process was observed for the material prepared by anchoring of Fc *via* formation of a Schiff base (Fc/NH_2_/SBA-15). During the second reaction cycle its reactivity was decreased from 64% (first cycle) to 23% (second cycle). In the third cycle, this material exhibited only negligible activity in CIP removal (11% of CIP conversion). In contrast to this sample, the stability of Fc/N_3_/SBA-15 and Fc/Cl/SBA-15 was found to be much higher (Fig. 10). Interestingly, the rate of deactivation of these two materials after each subsequent reaction cycle was almost the same, and after the third reaction cycle they still removed approximately 60% of the initial antibiotic pollutant (Fig. 10). In view of these results, one can clearly conclude that the anchoring of Fc on SBA-15 by the Friedel–Crafts alkylation and formation of triazole ring are better strategies for ensuring relatively high stability of these hybrid organic–inorganic materials in the photo-Fenton process than formation of a Schiff base.

In the majority of heterogeneous catalytic processes, deactivation of the catalysts under acidic conditions is related to leaching of the active components. In our study, Fc was anchored to SBA-15 supports by formation of various chemical bonds which are known for their different stability and resistibility to acidic conditions. For example, it is well-known that a Schiff base can easily hydrolyze under such reaction conditions,^43^ resulting in leaching of the anchored Fc from the catalyst. In order to verify whether the deactivation of catalysts prepared in this study was related to leaching of Fc or other phenomena (*e.g.* degradation of ferrocene^44,45^), additional experiments were performed. For this purpose, a given catalyst was stirred into a CIP solution (pH = 3) and agitated for 1 h under dark conditions. Then, the solid catalyst was separated by filtration and the concentration of the leached iron species in the filtrate was analyzed by ICP-OES. It was expected that this simple test will allow estimation of how much of the initial Fc was leached from the catalysts under such strongly acidic conditions. Surprisingly, the lowest percentage of the initial Fc species was leached from Fc/NH_2_/SBA-15 sample (Table S1†) which was characterized by the fastest deactivation rate in the photo-Fenton process after three reaction cycles (Fig. 10). In view of this information, one can conclude that the presence of both H_2_O_2_ and light in reaction media might play significant role in deactivation of the catalyst in which ferrocene was anchored *via* formation of a Schiff base. For Fc/N_3_/SBA-15 and Fc/Cl/SBA-15, the percentage of the initial Fc species leached from the catalysts under such acidic conditions was found to be slightly higher (21.6 and 28.4%, respectively; Table S1†), indicating lower acid resistibility of Fc species anchored *via* Friedel–Crafts alkylation and click reaction. Nevertheless, these two catalysts were found to be much more stable in a three subsequent reaction cycles than Fc/NH_2_/SBA-15 (Fig. 10). All these observations indicate that faster deactivation rate of the catalyst prepared by Schiff base formation may result, to some extent, from degradation or decomposition of the anchored Fc species under working conditions in the presence of H_2_O_2_ and light at pH = 3. This phenomenon will be discussed in details in further part of the article. Moreover, in view of comparable deactivation rate of Fc/N_3_/SBA-15 and Fc/Cl/SBA-15 catalysts (Fig. 10) and higher loading of Fc in the latter sample (Table 1), one can clearly conclude that Fc/N_3_/SBA-15 was the most stable from among all catalysts prepared in this study.

**Fig. 11:**
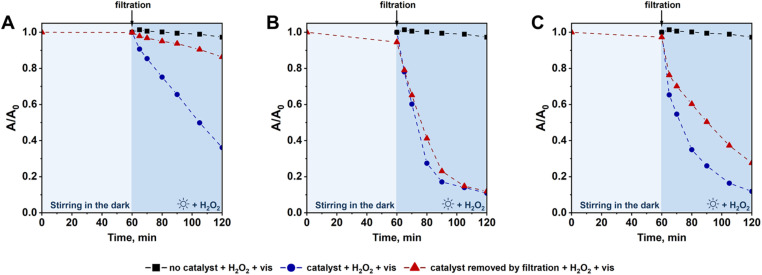
Contribution of Fc species leached from (A) Fc/NH_2_/SBA-15, (B) Fc/Cl/SBA-15, and (C) Fc/N_3_/SBA-15 after 1 h of stirring in the dark at pH = 3 to degradation of CIP *via* photo-Fenton process. For comparison purposes, efficiency of CIP degradation without catalyst and in the presence of a given heterogeneous catalyst are provided. Reaction conditions: in a given catalytic test evaluating reactivity of the leached ferrocene species, 30 mg of Fc-containing catalyst was stirred to CIP solution (80 mL, 15 mg L^−1^, pH = 3) and agitated in the dark for 1 h (stirring rate: 600 rpm). Then, the heterogeneous catalyst was separated by filtration and the filtrate was collected. In the next step, hydrogen peroxide (50 μL H_2_O_2_ (30%)) was added to the filtrate, and the photo-Fenton process was initiated by the exposure of the reaction mixture to visible light (*λ* > 400 nm).

As described above, Fc species anchored on all supports were leached during the photo-Fenton process. According to previous studies, dissolved ferrocene itself may efficiently activate H_2_O_2_ to form hydroxyl radicals.^46^ Thus, the question is what was the contribution of the leached ferrocene species to the overall degradation of the antibiotic during the first reaction cycle. To answer this question hydrogen peroxide was added to the filtrate obtained by separation of the heterogeneous catalysts from CIP solution after 1 h of stirring under dark conditions, and then the reaction mixture containing only dissolved (leached) ferrocene species (no heterogeneous catalyst) was exposed to visible light to initiate the photo-Fenton process. As shown in Fig. 11, the reactivity of Fc species leached from Fc/Cl/SBA-15 was almost the same as that observed in the presence of the heterogeneous catalyst. In view of this observation, one can clearly conclude that ferrocene species leached from Fc/Cl/SBA-15 have a significant contribution to H_2_O_2_ activation and CIP degradation during the first cycle of the photo-Fenton process. Of course, direct comparison of CIP degradation efficiency under such various reaction conditions (reaction with the use of heterogeneous catalyst *vs.* reaction in the presence of leached Fc species only) is not possible because of different intensification of the light scattering effect in the presence of the solid sample and different initial concentration of Fc species dissolved in reaction media at the beginning of the photo-Fenton process. However, important role of the leached ferrocene species in CIP degradation can be undoubtedly concluded based on these experimental data. In the case of Fc/N_3_/SBA-15, the contribution of the dissolved ferrocene to overall degradation of the antibiotic was lower, as expected from lower concentration of Fc in this sample and their higher resistibility to acidic conditions, but these species still played an important role in CIP removal (Fig. 11). In contrast to the above-mentioned samples, the reactivity of Fc species leached from Fc/NH_2_/SBA-15 was found to be significantly reduced when compared to the reaction with the use of the corresponding heterogeneous catalyst (reaction without separation of the catalyst; Fig. 11). Thus, only for sample Fc/NH_2_/SBA-15, the contribution of the leached ferrocene species to degradation of CIP was relatively low, and the main active component responsible for the antibiotic degradation were Fc species anchored to SBA-15 support. Very low activity of Fc species leached from Fc/NH_2_/SBA-15 after 1 h of stirring in the dark resulted more likely from their relatively low concentration in the reaction media (Table S1†). Possible degradation or decomposition of Fc species leached from this catalyst after stirring in the dark at pH 3 was excluded, since these dissolved species were even more reactive than those leached from Fc/N_3_/SBA-15 and Fc/Cl/SBA-15 catalysts (Fig. S11†). However, one cannot totally exclude that some of the Fc species anchored to Fc/NH_2_/SBA-15 may be degraded under working conditions (*i.e.* in the presence of H_2_O_2_ and light at pH = 3), resulting in the fastest deactivation rate of this catalyst in three subsequent reaction cycles. Detail explanation of this phenomenon requires additional studies which are underway.

Based on above data, one can conclude that even if Fc species were leached from all catalysts, they still acted as the active components in activation of H_2_O_2_*via* photo-Fenton process. A similar contribution of the dissolved ferrocene species to efficient degradation of methylene blue dye has been previously reported in reference.^46^ Thus, one can clearly conclude that in all samples prepared in this work, SBA-15 played a double role of: (i) a support on which the active component was immobilized and took part in H_2_O_2_ activation toward formation of strongly oxidizing hydroxyl radicals, and (ii) a reservoir from which the active Fc species were released during the reaction by their leaching from the catalyst surface to reaction media. The latter process was found to be the main factor contributing to deactivation of Fc-containing materials in three subsequent reaction cycles and its rate was strongly affected by the type of Fc anchoring method. As far as the reactivity of Fc species is concerned, it is also important to stress that the leached Fc species were found to be much more efficient in CIP degradation *via* the photo-Fenton process than bulk ferrocene (Fig. 8). Thus, even if SBA-15-based catalysts are not highly stable from a long-term perspective, the use of the heterogeneous catalysts in which Fc species are anchored on the surface of a siliceous support is more beneficial than the direct use of a significantly greater quantity of bulk ferrocene (Fig. 8).

As far as reactivity of Fc-containing catalysts synthesized in this work is concerned, it is important to underline that the most active material (*i.e.* Fc/Cl/SBA-15; *k* = 0.0629 min^−1^) was found to be more reactive than LaFeO_3_/diatomite (*k* = 0.0187 min^−1^),^47^ and Fe_3_O_4_-activated carbon composite (*k* = 0.0231 min^−1^)^48^ reported previously in literature (for more details please see Table S2†). Moreover, it exhibited comparable reactivity to that established for C_3_N_4_/Fe_3_O_4_/MIL-100(Fe) ternary heterojunction (*k* = 0.0656 min^−1^).^49^ However, reactivity of Fc/Cl/SBA-15 was found to be lower than that reported previously for rGO–ZnFe_2_O_4_ (*k* = 0.095 min^−1^) by Yang *et al.*^50^

## 4. Conclusions

Results obtained in this study clearly show that ferrocene anchoring method has a significant impact on the efficiency of Fc loading as well as the reactivity and stability of SBA-15-based catalysts. In terms of the efficiency of Fc anchoring, the highest ferrocene loading was achieved by the Friedel–Crafts alkylation, while the least efficient was the immobilization of Fc *via* formation of a Schiff base between the amino-organosilane and ferrocenecarboxyaldehyde. As concerns the reactivity of a single active site in the photo-Fenton process, the most reactive were ferrocene species from the catalyst prepared by formation of triazole ring (click reaction). It was also established that in all catalytic tests, the main ROS responsible for efficient degradation of CIP were hydroxyl radicals. However, the nature of the main active sites responsible for efficient activation of H_2_O_2_ toward formation of hydroxyl radicals was different for various catalysts. In the samples prepared by the click reaction and Friedel–Crafts alkylation, the role of leached ferrocene species in activation of H_2_O_2_ to form hydroxyl radicals was much greater than that observed for the catalyst prepared by Schiff base formation. It showed that the Fc anchoring method affects not only the efficiency of ferrocene anchoring and its reactivity, but also the catalysts stability. The highest deactivation rate in three subsequent reaction cycles was observed for the sample prepared by Schiff base formation. The catalysts prepared by the click reaction and Friedel–Crafts alkylation were found to be much more stable in three subsequent reaction cycles and exhibited similar deactivation rates. Based on above, it was established that the most promising method for the preparation of SBA-15-based catalysts for degradation of ciprofloxacin *via* the photo-Fenton process is the click reaction. Ferrocene species anchored *via* this method on the siliceous support are not only the most reactive but also exhibit the highest long-term stability under working conditions applied in this work.

## Conflicts of interest

There are no conflicts to declare.

## Supplementary Material

RA-013-D3RA00188A-s001
