# Supplementary material for: The influence of ferrocene anchoring method on the reactivity and stability of SBA-15-based catalysts in the degradation of ciprofloxacin via photo-Fenton process
Source: RSC Adv. 2023 Mar 14;13(12):8360–73. doi: 10.1039/d3ra00188a (PMC10012415; doi:10.1039/d3ra00188a)
Supplement: RA-013-D3RA00188A-s001 [file RA-013-D3RA00188A-s001.pdf]

Electronic Supplementary Material (ESI) for RSC Advances.
This journal is © The Royal Society of Chemistry 2023

Electronic Supporting Information

for

RSC Advances

The influence of ferrocene anchoring method on the reactivity and stability of SBA-15-based catalysts in degradation of ciprofloxacin via photo-Fenton process

*Adrian Walkowiak, Lukasz Wolski, and Maria Ziolek**

Adam Mickiewicz University, Poznań, Faculty of Chemistry, Uniwersytetu Poznańskiego 8, 61-614 Poznań, Poland

* Corresponding author

email: ziolek@amu.edu.pl

Table of contents:

Fig. S1 Pore sizes distributions determined by DFT method for the prepared materials.

Fig. S2 SEM images of pristine SBA-15 and siliceous support modified with organosilanes.

Fig. S3 EDS elemental mapping images of pristine SBA-15.

Fig. S4 EDS elemental mapping images of Fc/NH_2/SBA-15.

Fig. S5 EDS elemental mapping images of Fc/Cl/SBA-15.

Fig. S6 EDS elemental mapping images of Fc/N_3/SBA-15.

Fig. S7 Pseudo-first-order plot for determination of apparent CIP degradation rate via Fenton process in the presence of Fc-containing catalysts: (A) Fc/NH_2/SBA-15, (B) Fc/Cl/SBA-15, (C) Fc/N_3/SBA-15.

Fig. S8 Efficiency of CIP removal via photo-Fenton process in the presence of the parent SBA-15 and all functionalized supports used for ferrocene anchoring.

Fig. S9 Pseudo-first-order plot for determination of apparent CIP degradation rate via photo-Fenton process in the presence of Fc-containing catalysts: (A) Fc/NH_2/SBA-15, (B) Fc/Cl/SBA-15, (C) Fc/N_3/SBA-15.

Fig. S10 Efficiency of CIP degradation via photo-Fenton process in the presence of Cu^{2+} cations.

Fig. S11 Normalized efficiency of CIP degradation via photo-Fenton process in the presence of ferrocene species leached from the given heterogeneous catalysts after 1 h of stirring in the dark at pH = 3.

Table S1 The concentrations of metals (Fe, Zn, Cu) in the post-reaction mixture after 60 minutes of the photo-Fenton process in the presence of a given catalyst.

Table S2 A comparison of the reactivity of catalysts synthesized in this work with that reported previously for other nanomaterials in degradation of CIP via photo-Fenton process.

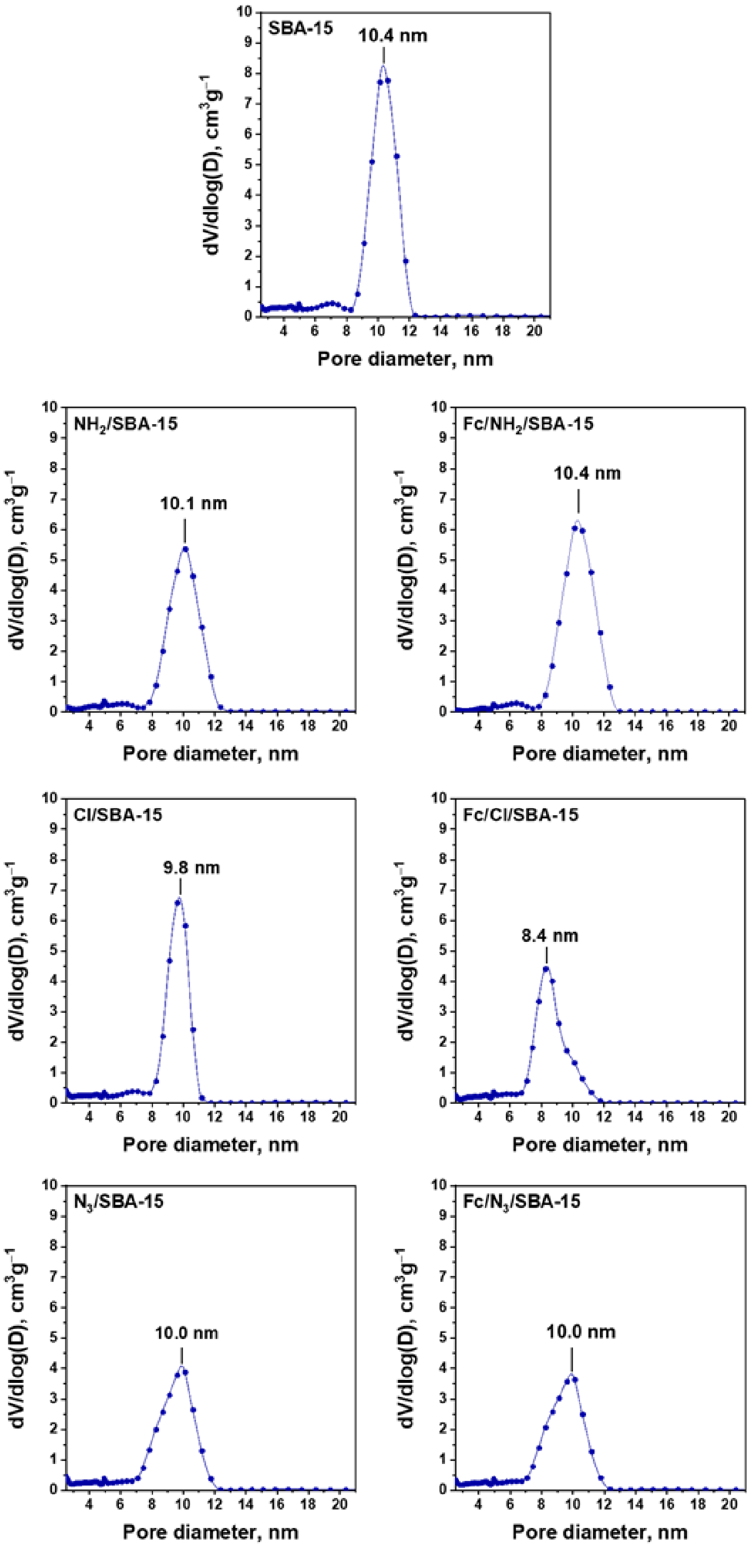

Fig. S1 Pore sizes distributions determined by DFT method for the prepared materials.

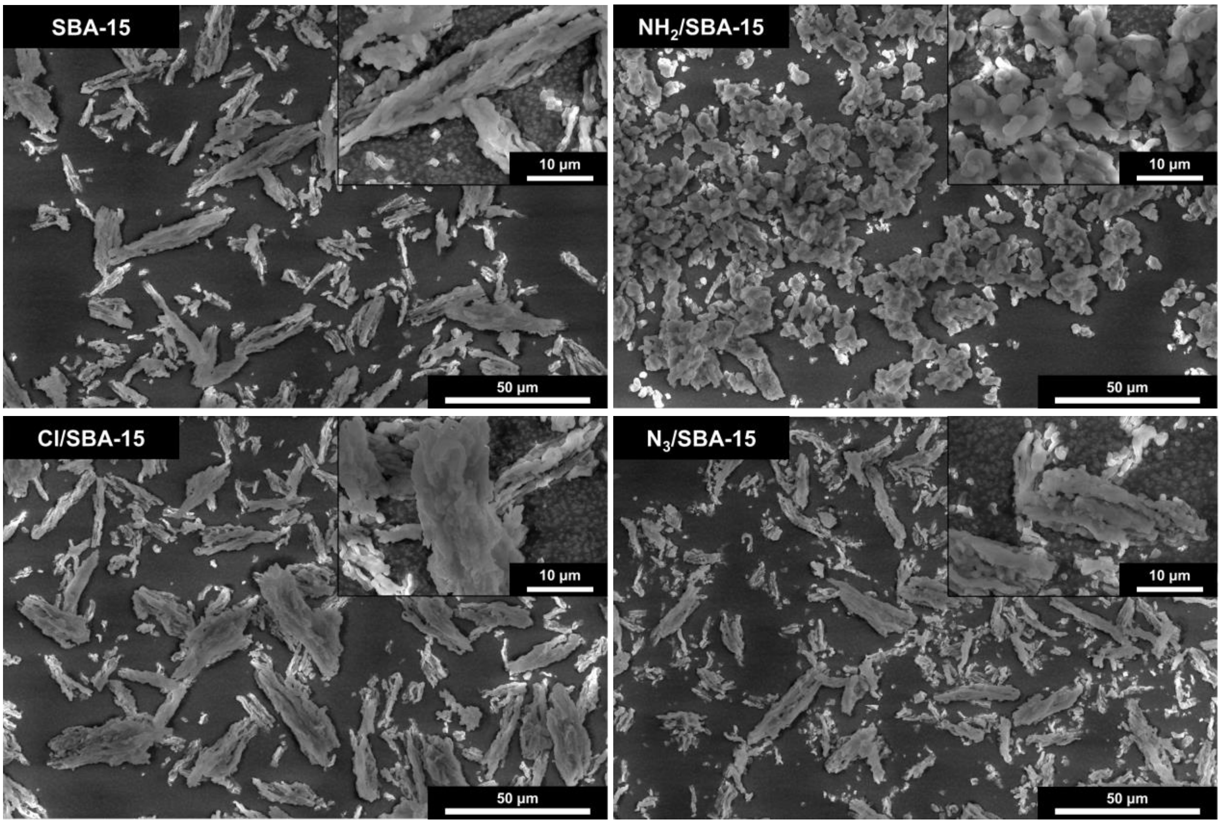

Fig. S2 SEM images of pristine SBA-15 and siliceous support modified with organosilanes.

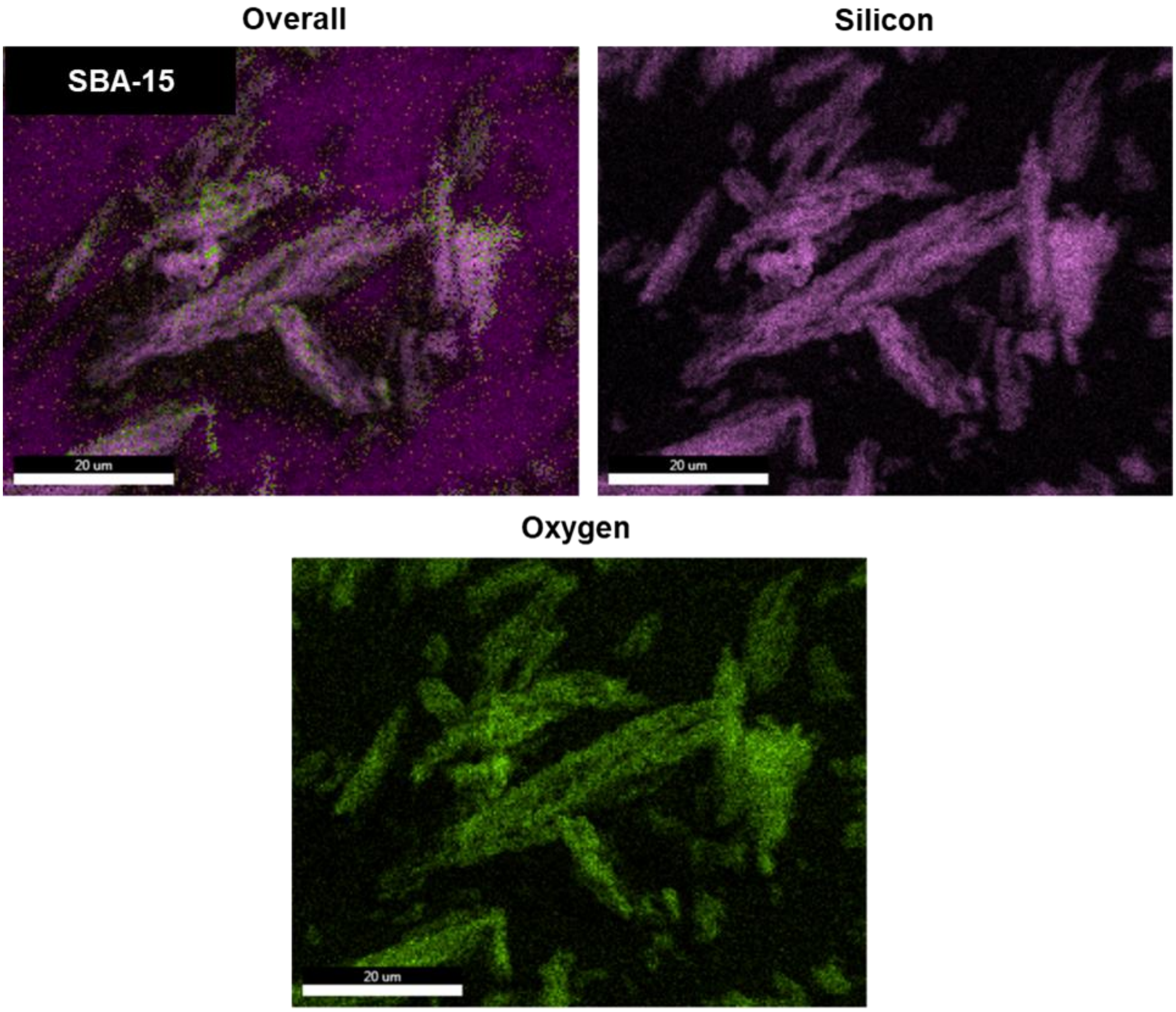

Fig. S3 EDS elemental mapping images of pristine SBA-15.

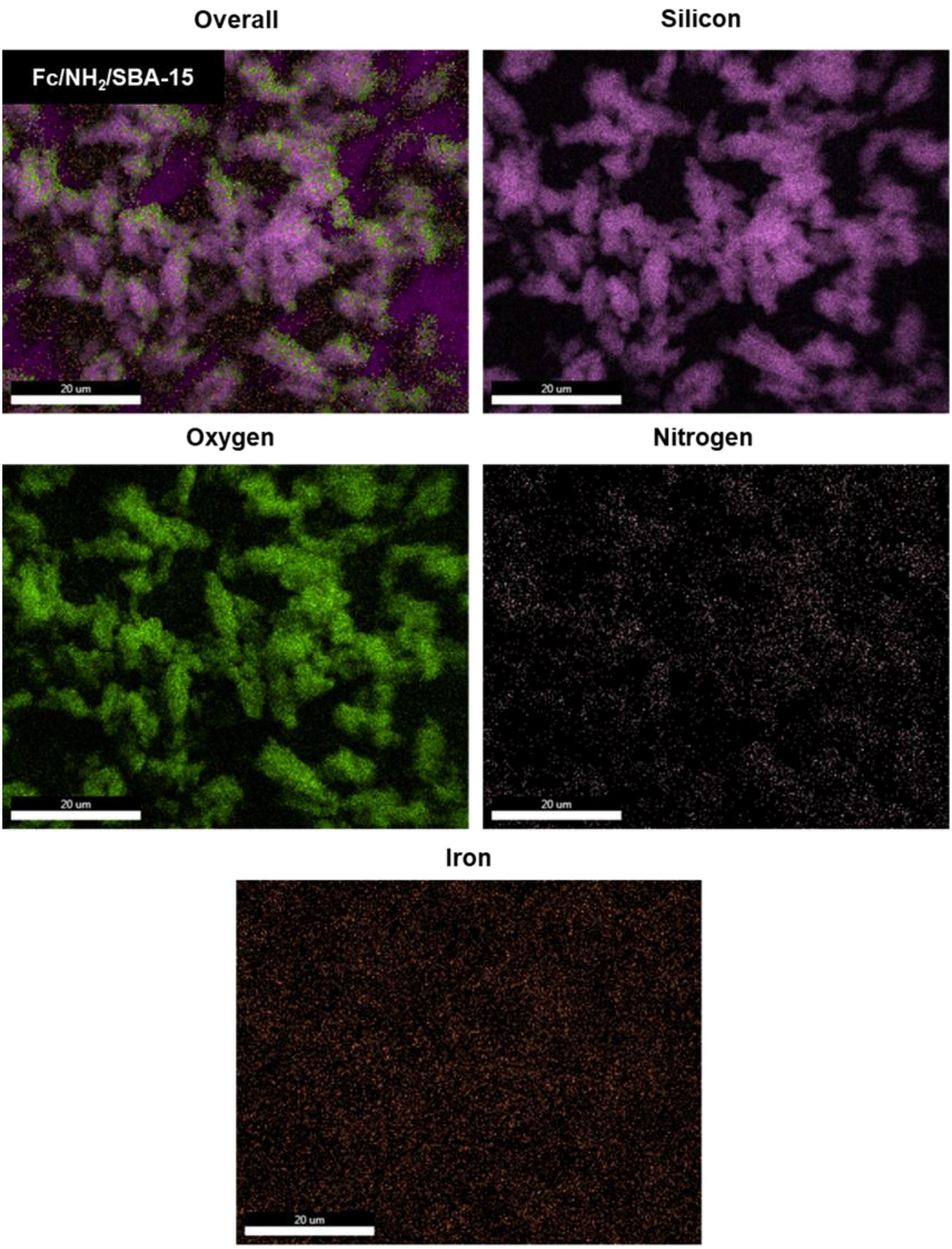

Fig. S4 EDS elemental mapping images of Fc/NH_2/SBA-15.

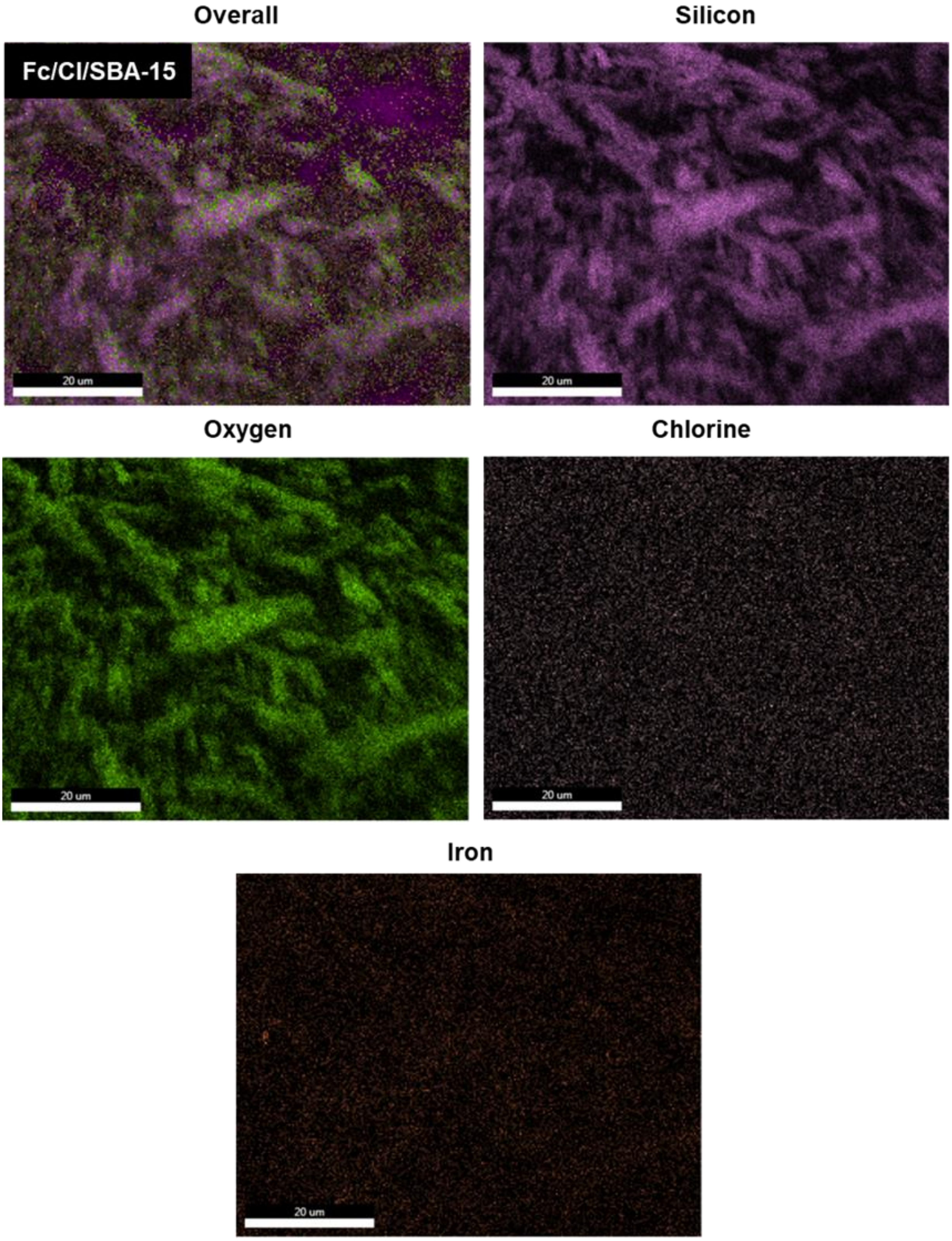

Fig. S5 EDS elemental mapping images of Fc/Cl/SBA-15.

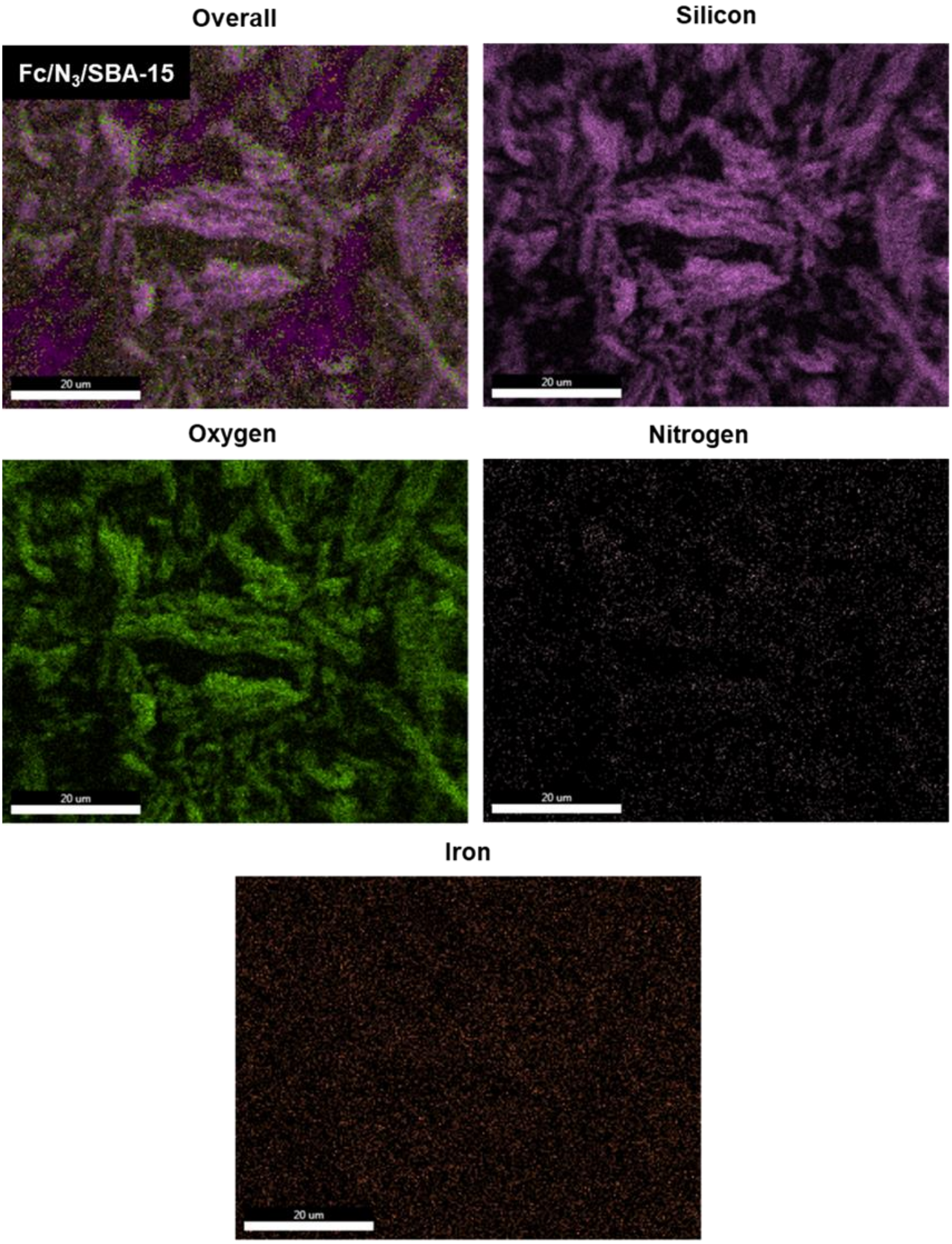

Fig. S6 EDS elemental mapping images of Fc/N_3/SBA-15.

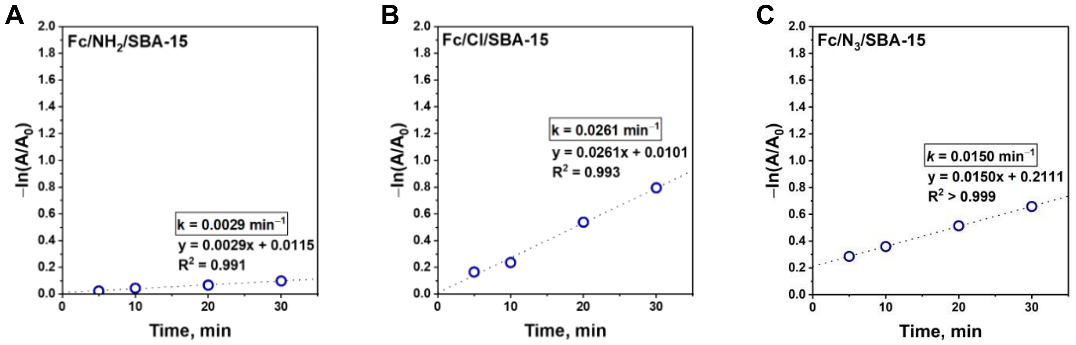

Fig. S7 Pseudo-first-order plot for determination of apparent CIP degradation rate via Fenton process in the presence of Fc-containing catalysts: (A) Fc/NH_2/SBA-15, (B) Fc/Cl/SBA-15, (C) Fc/N_3/SBA-15. The apparent reaction rate constant (*k*) is provided in the figure. *Reaction conditions*: 30 mg of the catalyst, 80 mL of CIP solution (15 mg/L, pH = 3), 50 μL of H_2O_2 (30%), stirring rate: 600 rpm. Reaction performed in the dark.

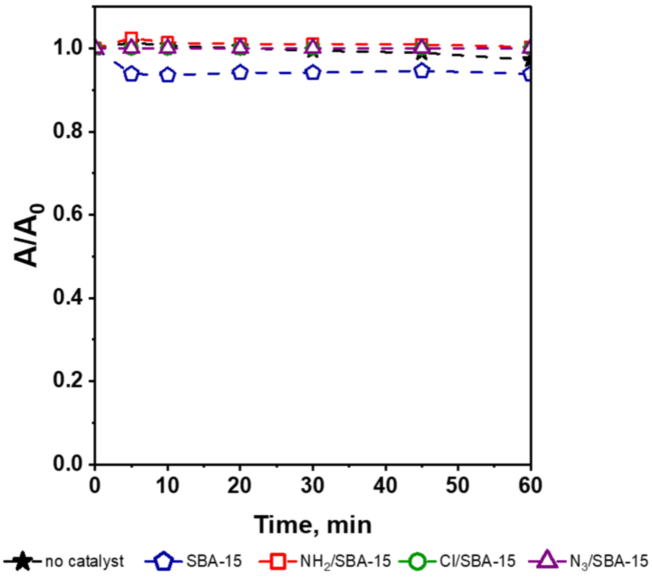

Fig. S8 Efficiency of CIP removal via photo-Fenton process in the presence of the parent SBA-15 and all functionalized supports used for ferrocene anchoring. *Reaction conditions*: 30 mg of the catalyst, 80 mL of CIP solution (15 mg/L, pH = 3), 50 μL of H_2O_2 (30%), irradiation with visible light (λ > 400 nm), stirring rate: 600 rpm.

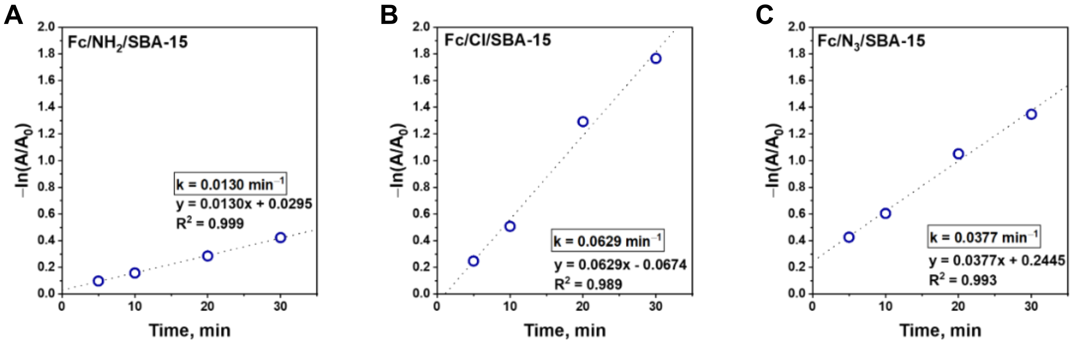

Fig. S9 Pseudo-first-order plot for determination of apparent CIP degradation rate via photo-Fenton process in the presence of Fc-containing catalysts: (A) Fc/NH_2/SBA-15, (B) Fc/Cl/SBA-15, (C) Fc/N_3/SBA-15. The apparent reaction rate constant (*k*) is provided in the figure. *Reaction conditions*: 30 mg of the catalyst, 80 mL of CIP solution (15 mg/L, pH = 3), 50 µL of H_2O_2 (30%), irradiation with visible light ($\lambda > 400$ nm), stirring rate: 600 rpm.

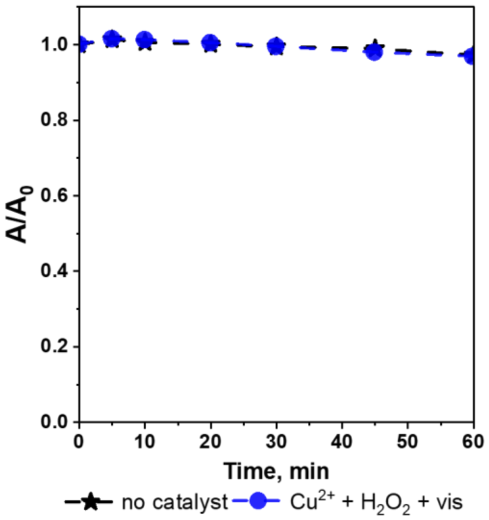

Fig. S10 Efficiency of CIP removal via photo-Fenton process in the presence of Cu^{2+} cations. *Reaction conditions*: 50 μL of the Cu^{2+} solution (16 mM), 80 mL of CIP solution (15 mg/L, pH = 3), 50 μL of H_2O_2 (30%), irradiation with visible light (λ > 400 nm), stirring rate: 600 rpm. The amount of added Cu^{2+} cations was comparable to the amount of copper species in 30 mg of Fc/N_3/SBA-15 sample.

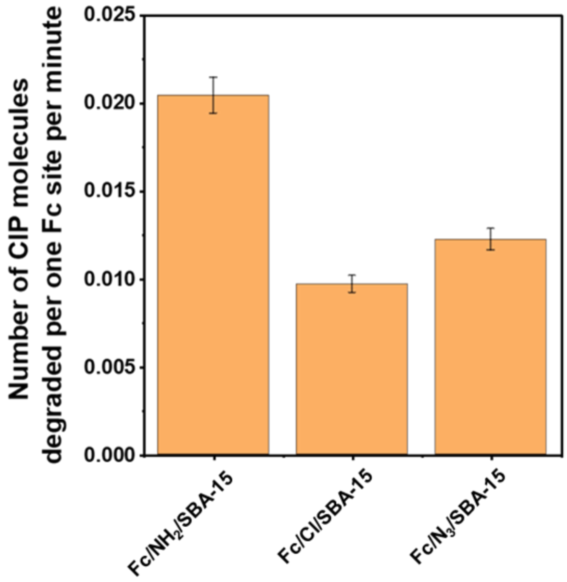

Fig. S11 Normalized efficiency of CIP degradation via photo-Fenton process in the presence of ferrocene species leached from the given heterogeneous catalysts after 1 h of stirring in the dark at pH = 3. In a given catalytic test, 30 mg of Fc-containing catalyst was stirred into the CIP solution (15 mg/L, pH = 3) and agitated in the dark for 1 h. Then, the heterogeneous catalyst was separated by filtration and the filtrate was collected. In the next step, hydrogen peroxide was added to the filtrate, and the photo-Fenton process was initiated by the exposure of the reaction mixture to visible light for 30 min. The reactivity of the leached ferrocene species was expressed as number of CIP molecules degraded by ROS formed on a single Fc site per time unit. *Reaction conditions*: 80 mL of CIP solution (15 mg/L, pH = 3) containing the leached ferrocene species, 50 μL of H_2O_2 (30%), irradiation with visible light ($\lambda > 400$ nm), stirring rate: 600 rpm.

Table S1 The concentrations of metals (Fe, Zn, Cu) in the post-reaction mixture after 60 min of stirring CIP solution at pH = 3 in the dark in the presence of a given catalyst. The percentages of leached elements were calculated based on ICP-OES analyses and provided. *Reaction conditions:* 30 mg of the catalyst, 80 mL of CIP solution (15 mg/L, pH = 3), stirring rate: 600 rpm. After 60 minutes of the reaction, the heterogeneous catalyst was separated by filtration through syringe filter (0.2 μm), and the filtrate was analyzed by means of ICP-OES.

Catalysts	Fe[a] [ppb]	% of leached Fe	Zn[a] [ppb]	% of leached Zn	Cu[a] [ppb]	% of leached Cu
Fc/NH_2/SBA-15	255	15	<1	–	<1	–
Fc/Cl/SBA-15	1682	28	104	92	<1	–
Fc/N_3/SBA-15	632	22	<1	–	641	~100%
no catalyst	1	–	<1	–	<1	–

Table S2. A comparison of the reactivity of catalysts synthesized in this work with that reported previously for other nanomaterials in degradation of CIP via photo-Fenton process.

No.	Catalyst symbol	Initial CIP concentration [mg/L]	Catalyst loading [mg/L]	Initial H_2O_2 concentration* [mmol/L]	Light source	pH of the reaction mixture	Efficiency of CIP elimination	Reaction rate constant [min^{-1}]	Ref.
1	Fc/NH_2/SBA-15	15	375	6.1	Visible light (Hg-Xe lamp equipped with with a 400 nm cut-off filter)	3	64% after 60 min of reaction	0.0130	This work
2	Fc/Cl/SBA-15	15	375	6.1	Visible light (Hg-Xe lamp equipped with with a 400 nm cut-off filter)	3	90% after 60 min of reaction	0.0629	This work
3	Fc/N_3/SBA-15	15	375	6.1	Visible light (Hg-Xe lamp equipped with with a 400 nm cut-off filter)	3	89% after 60 min of reaction	0.0377	This work
4	$LaFeO_3$/Diatomite	20	300	291	Visible light (Xe lamp)	4	99% after 150 min of reaction	0.0187	[1]
5	rGO-$ZnFe_2O_4$ composites (7 %rGO-$ZnFe_2O_4$)	10	300	20	Polychromatic UV-vis light (Xe lamp)	4	~100% of CIP degradation in 40 min of reaction	0.095	[2]
6	Fe_3O_4-activated carbon composite (AC4/F)	–	1500	10	Polychromatic UV-vis light (Hg lamp)	2	~100% of CIP degradation in 120 min of reaction	0.0231	[3]
7	C_3N_4/Fe_3O_4/MIL-100(Fe) ternary heterojunction	200	670	78	Visible light (420 nm cut-off filter)	3	95.3% of CIP removal after 150 min of the reaction	0.0656	[4]

[1] Y. Liu, M. Wu, L. Ren, C. Wang, Z. Li, L. Ling, Y. Guo, ChemistrySelect 5 (2020) 14792–14799.

[2] L. Yang, Y. Xiang, F. Jia, L. Xia, C. Gao, X. Wu, L. Peng, J. Liu, S. Song, Appl. Catal. B Environ. 292 (2021) 120198.

[3] A.B. Fortunato, F.E. Bimbi Júnior, J.M. Moreira, T.S.B. de Barros, A.L. de J. Pereira, M.A.G. Trindade, T.A.D. Colman, P.H. Suegama, W.R.P. Barros, F. Gozzi, C.T. de Carvalho, J. Water Process Eng. 50 (2022) 103263.

[4] W. He, H. Jia, Z. Li, C. Miao, R. Lu, S. Zhang, Z. Zhang, J. Environ. Chem. Eng. 10 (2022) 108698.
